# Epigenetics as a Mechanism of Developmental Embodiment of Stress, Resilience, and Cardiometabolic Risk Across Generations of Latinx Immigrant Families

**DOI:** 10.3389/fpsyt.2021.696827

**Published:** 2021-07-20

**Authors:** Elizabeth S. Clausing, Amy L. Non

**Affiliations:** Department of Anthropology, University of California San Diego (UCSD), La Jolla, CA, United States

**Keywords:** epigenetic, embodiment, stress, resilience (psychological), Latinx, maternal and child health, cardiometabolic health

## Abstract

Psychosocial stressors can become embodied to alter biology throughout the life course in ways that may have lasting health consequences. Immigrants are particularly vulnerable to high burdens of stress, which have heightened in the current sociopolitical climate. This study is an investigation of how immigration-related stress (IRS) may impact the cardiometabolic risk and epigenetic markers of Latinx immigrant mothers and children in Nashville, TN. We compared stress and resilience factors reported by Latina immigrant mothers and their children (aged 5–13) from two time points spanning the 2016 U.S. presidential election (June 2015–June 2016 baseline, *n* = 81; March–September 2018 follow-up, *n* = 39) with cardiometabolic risk markers (BMI, waist circumference, and blood pressure). We also analyzed these factors in relation to DNA methylation in saliva of stress-related candidate genes (*SLC6A4* and *FKBP5*), generated *via* bisulfite pyrosequencing (complete case n's range from 67–72 baseline and 29–31 follow-up) (n's range from 80 baseline to 36 follow-up). We found various associations with cardiometabolic risk, such as higher social support and greater acculturation were associated with lower BMI in mothers; discrimination and school stress associated with greater waist circumferences in children. Very few exposures associated with *FKBP5*, but various stressors associated with methylation at many sites in *SLC6A4*, including immigrant-related stress in both mothers and children, and fear of parent deportation in children. Additionally, in the mothers, total maternal stress, health stress, and subjective social status associated with methylation at multiple sites of *SLC6A4*. Acculturation associated with methylation in mothers in both genes, though directions of effect varied over time. We also find DNA methylation at *SLC6A4* associates with measures of adiposity and blood pressure, suggesting that methylation may be on the pathway linking stress with cardiometabolic risk. More research is needed to determine the role of these epigenetic differences in contributing to embodiment of stress across generations.

## Introduction

The accumulation of stress over time can contribute to biological embodiment across the life course. Stressors in the early life environment may influence susceptibility to later life chronic disease. Latinx Americans, regardless of immigration status, often experience high rates of chronic stress and are at risk for high rates of cardiometabolic disease later in life, including obesity and hypertension. For example, Latinx adults have 1.65 times the rate of diabetes (1, 2) and 1.2 times the rate of overweight and obesity, relative to non-Latinx whites (3). These health disparities often emerge early in life: overall Latinx children aged 2–19 have 1.6 times the rate of obesity as white children (1), and the youngest group of Latinx children aged 2–5 years have *nearly quadruple* the rates of obesity as white children in the US (4, 5). Currently, immigrants and their US-born children comprise 85.7 million people, which is about 26 percent of the overall US population (6), and nearly half of which report Latinx origins. Thus, Latinx Americans represent an important demographic for the health of the future of the US population.

Latinx immigrant families may be particularly at risk for stress-related diseases, such as cardiometabolic diseases like hypertension and diabetes, as they experience high rates of stressors related to immigrant experiences, including legal status, uncertain immigrant policies, and acculturative stress (7–9). Children of recent immigrants may be at even higher risk than their first-generation immigrant parents, as health outcomes tend to worsen with longer duration in the US, and risk increases across generations, suggesting that racism, xenophobia, or poor living conditions in the US lead to worse health, rather than immigration itself (10–12). Regardless of their own immigrant status, children of Latinx immigrants are disadvantaged on numerous fronts, including access to quality education, health care, and job security for their parents, while being confronted at the same time with resentment, racism, and often violence. Additionally, Latinx children and their parents face heightened stereotypes resulting from recent and highly publicized ongoing debates on immigration policy regarding the Mexican border. These social determinants can affect immigrant families' lifestyles, risk of disease, and mortality. For example, studies find associations between racial discrimination and higher BMI, waist circumference, and blood pressure in young children and in adults (13, 14). While changes in diet and exercise likely contribute to these outcomes, stressors may also become embodied directly through molecular mechanisms that may influence lifetime risk of cardiometabolic disease (15–17).

Embodiment, in this paper, refers to the process by which one's life experiences are literally incorporated biologically, at a molecular level, to influence later life health and disease (18). This process of embodiment is in contrast to the notion of genetic determinism, in which one's phenotype is permanently set by inherited genes. Epigenetic mechanisms, such as DNA methylation, are one process of embodiment by which an individual's phenotype can change over his/her lifetime in response to the environment. The epigenome may be particularly sensitive to embodiment in early life environments, or critical periods, when biological systems are actively being developed (19).

While early life may be the most sensitive period for epigenetic processes, the accumulation of experience over a life course can also alter the epigenome, through potentially environmentally influenced or stochastic changes, as evidenced by identical twins reared apart (20, 21) and aging studies (22–24). When environmentally influenced, epigenetic changes may be a mechanism for the process of weathering, whereby the cumulative effect of social disadvantage over time, such as poverty or discrimination, disproportionately affects the health of disadvantaged populations (25, 26). Regardless of the time period of exposure, the epigenome is clearly sensitive to environmental forces, and has been theorized to help explain the persistence of racial and social health disparities, such as the increased burden of cardiovascular disease among Black relative to white Americans (27), and the higher rates of chronic diseases, such as obesity and diabetes, among Latinos (28). However, as most prior research only examines one time point for epigenetic measures, usually early in childhood, we do not yet know how the epigenome responds to environmental exposures across the lifespan, or how much it contributes to racial disparities in cardiometabolic health.

Most social epigenomic studies to date focus on DNA methylation, which is the addition of a methyl group to the 5′ carbon of a cytosine. Many of these studies tend to use a hypotheses-free genome-wide approach, relying on preselected sites on a microarray, which tend to miss important gene regions (29). Targeted approaches can be useful extensions on this work, when specific hypotheses exist for certain genes, and when working with smaller sample sizes. For example, there are two genes well-established to be involved in the regulation of stress response, including the glucocorticoid receptor chaperone protein gene FK506 Binding protein 5 (*FKBP5*), which is an important regulator of glucocorticoid receptor sensitivity (30, 31) and the serotonin transporter gene (*SLC6A4*), which has been implicated in stress response and increased risk for psychiatric disorders (32). Both genes regulate stress response, and also have a potential role in influencing cardiometabolic risk (15–17).

*FKBP5* is a gene known to regulate the glucocorticoid receptor, an important part of the hypothalamic pituitary adrenal (HPA) axis that binds cortisol—an adrenal stress hormone that also regulates metabolism—and thus a good candidate to mediate an epigenetic response to social stress. Many studies have identified associations between adverse childhood experiences, socioeconomic adversity, and other environmental stressors with lower DNA methylation of *FKBP5* across various tissues as reviewed in Parade et al. (33). Demethylation of intron 2 of *FKBP5* has been shown to disrupt the HPA axis and contribute to glucocorticoid resistance, higher cortisol levels, and prolonged recovery following exposure to stress (34). One study of maternal-neonatal pairs in the Democratic Republic of Congo found significant methylation changes at different transcription factor binding sites in *FKBP5*, along with other HPA axis related genes, in association with chronic stress and war trauma across maternal blood, placenta, and newborn cord blood (35). Decreased methylation at *FKBP5* has also been identified in blood cells of adults who had experienced childhood trauma (36), in blood cells of Holocaust survivors with Post-Traumatic Stress Disorder (PTSD) and their offspring (37), and in buccal epithelial cells of adolescents who experienced early-life institutionalization in Romania (38). One study found methylation in one site of *FKBP5* was a predictor of both PTSD symptom severity and resilience (in opposite directions) in saliva (39). However, a different study found an opposite direction of effect in saliva, such that stress in childhood was associated with *higher* methylation of this gene in young adulthood (40), and a number of studies found no associations between childhood adversity and methylation at this gene (41–43) (though methylation and expression of this gene were usually linked with higher rates of depression) (41, 42). Higher methylation at this gene has also been linked with less healthy metabolic measures, such as waist circumference (15). While there is some inconsistency in findings, the majority of studies observed significant associations of child adversity and methylation across tissues, suggesting systemwide effects of early adversity on methylation at this gene, which may have relevance for cardiometabolic risk.

*SLC6A4* is a second gene with well-established associations with maternal care, early life adversity, and long-term child development. This gene is involved in serotonin and dopamine releases and has been implicated in stress response and increased risk for psychiatric disorders (44). *SLC6A4* helps regulate the bioavailability of serotonin and thus modulates mood, anxiety, and energy homeostasis. The majority of studies investigating this gene found that the 5-HTTLPR genotype moderates the relationship between early life stress and depression (45, 46). A growing number of studies have also found DNA methylation in the promoter of this gene in blood to be positively associated with child adversities (47–50), though in contrast one study found *decreased* promoter methylation in maternal and cord blood associated with depression in pregnancy (51). Methylation at *SLC6A4* also has been related to cardiometabolic health, as one study found promoter hypermethylation in blood leukocytes to be significantly associated with an increased prevalence of obesity (16). Altered methylation of CpG5 in cord blood was also found to be associated with greater concurrent measures of adiposity including BMI and waist circumference (17).

Latinx immigrant families in the US are living in a state of chronic stress, constantly on edge with concerns about deportation of their family members, or their ability to remain in the country in which most of them have lived for the majority of their lives, particularly in the years since the 2016 Presidential election. The epigenetic effects of increasing stress and anxiety associated with this particular sociopolitical moment for Latinx families are yet to be studied. To our knowledge, only one other study, completed in 2010, has investigated associations between discrimination and DNA methylation in Latina women (52). In blood from 147 pregnant Latina women, they identified decreased methylation in *FKBP5* associated with increased discrimination over time, though not always in expected directions. Only our prior study has investigated epigenetics of children of immigrants in relation to multiple psychosocial stress and resilience factors (53). We generally found increased stress and decreased social support associated with decreased epigenetic age, suggesting stress may slow child development. Few other studies have examined epigenetics of social support or any other positive social factor.

### Study Purpose and Hypotheses

The current study examined associations between psychosocial stressors and resilience factors with cardiometabolic risk factors, along with targeted DNA methylation in the *FKBP5* and *SLC6A4* genes in saliva of Latina immigrant mothers and their children. This study represents a targeted extension of our previous genome-wide analysis of the children's samples (53). We hypothesized that stressors measured both in childhood and adulthood would be associated with cardiometabolic markers including higher BMI, waist circumference, and blood pressure. We further predicted that stressors would associate with decreased DNA methylation at key CpG sites within intron 2 of *FKBP5*, and with increased methylation of the promoter region of *SLC6A4*. Finally, we predicted that methylation at these genes may associate with cardiometabolic markers. Overall, this study may be the first to examine repeated epigenetic and cardiometabolic measures in relation to stress and resilience within a longitudinal sample of Latina immigrant mothers and their children.

## Materials and Methods

### Study Population

The study sample draws from a longitudinal study of stress embodiment, entitled “Children of Hispanic Immigrants Collaborating to Overcome Stress” (CHICOS). The study recruited 81 families (mother-child dyads) in Nashville, TN between June 2015 and June 2016. This initial time point is called “baseline” throughout the rest of the study. Participants were recruited from local immigrant-serving community centers with subsequent snowball sampling. Inclusion criteria were self-described Latina, foreign-born immigrant mothers above age 18, with a child between the ages of 5–13. The mother and focal child were assessed on a number of different biological, psychosocial, and health-related measures. Following baseline assessment of the dyads, we revisited the same families 2–3 years later (March–September 2018), collecting the same data and new interviews focused on changes since the 2016 presidential election, from all available mothers and children who participated in the baseline sample (*n* = 39). This time point is referred to as “follow-up” throughout the study. Informed oral consent was provided by all participants, and Vanderbilt University and University of California San Diego Institutional Review Boards approved all protocols. All data are available upon request, and key measures are available in Supplementary Table 4.0.

### Exposures: Psychosocial Stressors and Resilience Factors

All exposure data were collected using surveys administered in person with mothers in Spanish (1.5–2 h) and children primarily in English (45 min). Surveys were a combination of validated scales and new survey questions developed following a set of preliminary individual interviews in 2014 with children, and focus groups with Latina immigrant mothers in Nashville, TN (54). Questions included in each scale are described in brief below, and detailed further elsewhere (53).

#### Psychosocial Stressors

Multiple measures of psychosocial stress were assessed, covering a range of domains, and including maternal and child assessment of each domain. Measures reported at both time points separately by children and mothers included *immigrant-related stress (IRS), discrimination stress*, and a *total stress score*. Measures additionally reported by only mothers included *family economic stress, family health stress*, and *household stress*. A measure reported only by the children was *school stress*. The questions included in each stress scale and Cronbach's alpha score for internal reliability are available elsewhere (53). The validated e*veryday discrimination* scale was also reported separately for mothers and children at the follow-up time point (55). This scale has been validated in Latina adults (56). All indices were calculated by taking the mean of responses for those not missing more than two questions, and higher scores indicate more stress across all measures. Outside of the scales, we also examined one individual question about the *child's fear of parent deportation*.

#### Resilience Measures

Measures of resilience included *social support* and *optimism* reported separately by mothers and children. We developed child-focused social support questions focusing on support from the parents (53). Social support and social connection in mothers were measured with a modified index from the Berkman Syme Social Network Index (57). Generalized dispositional optimism was measured in the mothers using the revised 10-item version of the Life Orientation Test (58), a scale which has been validated in Latina adults (59). To measure optimism in the children, we used the Y-LOT, the youth version of the LOT-R, which has been validated in a racially diverse set of 3rd to 6th grade children (60). Y-LOT was only measured at follow-up.

A measure of *subjective social status* (SSS) was also considered a resilience factor, as higher scores indicate better social standing. SSS has been linked with self-reported health among immigrant Latinas, and has been hypothesized to capture immigrant experiences that may alter perceived self-worth (61). Maternal SSS was measured at baseline and follow-up time points with the MacArthur SSS scale, which asks mothers to report where they felt they fit on a social ladder (range 1–10) in relation to others in the US (62). This scale has been validated and demonstrated reliability among diverse racial/ethnic groups, including Latinas (63). We also used the language use subscale of the Short Acculturation Scale for Hispanics (SASH), which has shown to be highly correlated with the overall SASH scale in validation studies (64).

### Outcomes: Cardiometabolic Biomarkers and DNA Methylation

#### Cardiometabolic Biomarkers

Multiple measures of cardiometabolic risk were assessed in both the children and the mothers at both time points. In the children, we measured adiposity with Body Mass Index (BMI) percentile (adjusted for child age and gender) and waist circumference. In the mothers, we measured BMI, systolic blood pressure (SBP), and diastolic blood pressure (DBP).

#### DNA Methylation

Genomic DNA was extracted from 79 Oragene saliva samples from children in 2015–2016 and again in 2018 (38 samples) using standard protocols (DNA Genotek, Ottawa, Ontario, Canada). Genomic DNA was also extracted from saliva of 80 mothers at baseline and 40 mothers in follow-up. Saliva was stored at room temperature, per manufacturer's recommendation until DNA extraction. DNA was isolated from 500 μl of children's saliva using prepIT-L2P (Zymo Research, CA, USA) and stored at −20°C until time of analysis. DNA was excluded from four child samples at baseline and one child and two maternal samples collected follow-up due to low quality/concentration of DNA, as measured by Nanodrop and Qubit. The level of DNA methylation was assessed *via* bisulfite pyrosequencing at two CpG sites within intron 7 of the *FKBP5* gene and six sites within the promoter region of the *SLC6A4* gene. In the end, we generated high quality methylation data from 78 child and maternal samples at baseline and 36 child and 38 maternal samples at follow-up for *FKBP5*. For *SLC6A4*, we generated data for 78 child and 80 maternal samples at baseline and 37 samples for both groups at follow-up. These gene regions were chosen based on prior studies highlighting their importance in early life adversity in humans, other primates, and rodents (38, 65, 66). These specific CpG sites assayed were in important regulatory regions, including the glucocorticoid response element of intron 7 of *FKBP5*, and the promoter region of *SLC6A4*. Primers for *FKBP5* were designed by EpigenDx (Assay ADS3828-FS2), which covers two sites in Bin 2 of intron 7 (37). Specific primer information of these gene regions can be found in Supplementary Table 2.0.

In brief, 500 ng of DNA from each sample was bisulfite converted in duplicate using the EZ DNA Methylation Gold Kit (Zymo Research, CA), according to manufacturer's protocol. Duplicate bisulfite-converted DNA was mixed with 0.2 μM of each primer and amplified using the HotstarTaq plus Master Mix (Qiagen, CA). For each sample, PCRs were performed on each of the duplicate bisulfite treatments using the following protocol for *SLC6A4* gene: one cycle of 95°C for 5 min, 45 cycles of 94°C for 1 min, 58°C for 1 min, 72°C for 1 min, and 72° for 10 min. For *FKBP5* gene: one cycle of 95°C for 5 min, 45 cycles of 95°C for 30 s, 56°C for 30 s, 72°C for 30 s, and 68° for 10 min.

DNA methylation levels for all CpG sites were assessed using the Pyromark Q24 pyrosequencer, following standard protocols (Qiagen, CA). A bisulfite conversion check was included in each assay to verify full conversion of the DNA. If the difference between two bisulfite replicates exceeded two standard deviations (SDs) of the variation in the entire study population, a third bisulfite treatment was tested and the average of the two closest results was used. The final analytical sample with complete data at baseline for children ranged from 67 to 68, and for mothers *n* = 72, and follow-up children's sample *n* = 29–31, and mother's sample *n* = 30–31.

### Covariates

Mothers self-reported at each time point their own age, marital status, number of years lived in the US, legal status, and maternal smoking status. Children reported their own age and gender at each time point.

### Data Analyses

We first tested correlations between each psychosocial stressor and resilience factor, key demographic factors, and all measures of cardiometabolic risk, and DNA methylation across all studied sites of *SLC6A4* and *FKBP5*, separately at each time point, using Pearson's correlations, to guide model building in subsequent analyses.

We next modeled associations between each psychosocial stressor and resilience factor with all cardiometabolic risk markers, and separately with each methylation site, and the average across sites, in the two genes. While a formal mediation analyses was not supported by our small sample size, we also tested if the methylation levels at any site within either gene were associated with the cardiometabolic risk markers to test the hypothesis that methylation of stress-related genes may be on the pathway toward cardiometabolic disease.

All associations were modeled using linear regressions by adjusting for a set of key covariates, including child's age, mother's age, child's gender, and maternal smoking, which are known confounders in epigenetic studies (67). All associations are presented as Beta estimate (standard deviation), *p*-value. We tested additionally including socioeconomic and immigrant-related demographic factors, including maternal years in the US, legal status, marital status, and education level, but missing data across variables limited the value of these models, which are not presented. Where significant, we also report the effects of each of these factors given their role in contributing to the immigrant context. We chose not to adjust for multiple testing corrections, since these adjustments can be overly conservative for exploratory analyses, particularly when methylation sites are highly correlated within regions, and exposures are also correlated. All analyses were conducted in R (http://www.R-project.org).

In a secondary set of analyses, we examined differences in methylation levels over time in both mothers and children, as well as the overall relationship between mothers and children's methylation at each time point using paired *t*-tests. We also explored longitudinal changes in methylation with linear mixed models, adjusting for the same key demographics as other described models.

## Results

### Demographics

#### Population Characteristics

Demographic characteristics of our analytical sample of 79 baseline and 39 follow-up CHICOS participant mothers and children are displayed in Table 1. In brief, at baseline, mean age of children was 8.7 years, 56% female, and the majority were born in the US (72%). Mothers were mean age 34.6 years, primarily non-smokers (97.4%), mostly born in Mexico (86.8%), lived in the US on average 12.6 years, and majority self-reported undocumented (85.3%). Mothers were mostly married (85.5%) and with few years of education (mean 9 years), and over a third had trouble paying basic bills (39.5%). In comparing demographics of analyzed subjects vs. those lost to follow-up, we found those lost were significantly more likely to be undocumented and unmarried mothers at baseline, but did not differ in other measured demographics.

**Table 1:** Demographics, social stressors, and resilience factors over time, complete case data.

	**Baseline** **(*n* = 79)** ***n*, % or mean (sd, range)**	**Follow-up** **(*n* = 39)** ***n*, % or mean (sd, range)**	**Missing data** **baseline/follow-up**	***p*-value^**†**^**
**Child demographics**				
Age in years	8.67 (2.13, 5–13)	10.5 (2.10, 6–15)	1/2	** <0.001**
Gender (female)	44 (56.4%)	17 (47.2%)	1/1	–
Country of birth			0/2	–
United States	57 (72.2%)	27 (73.0%)		
Other	22 (27.8%)	10 (27.0%)		
**Mother demographics**				
Age in years	34.61 (5.95, 23–52)	37.45 (4.84, 29–47)	3/0	** <0.001**
Country of birth			3/1	–
Mexico	66 (86.8%)	35 (92.1%)		
Other Latinx country	10 (13.2%)	3 (7.9%)		
Years in US	12.59 (3.97, 4–27)	14.98 (3.46, 6.67–22.42)	4/2	** <0.001**
Legal status			4/3	** <0.001**
Undocumented	64 (85.3%)	33 (91.7%)		
Documented	11 (14.7%)	3 (8.3%)		
Marital status			3/1	**0.041**
Married	65 (85.5%)	29 (76.3%)		
Single	11 (14.5%)	9 (23.7%)		
Years of education	9.39 (3.18, 2–18)	9.57 (3.24, 2–16)	3/1	–
Trouble paying basic bills (yes)	30 (39.5%)	10 (27.0%)	3/2	0.228
Smoking frequency	2 (2.6%)	3 (7.9%)	3/1	0.617
**Child psychosocial stressors**				
Total child stress (0–2)	0.56 (0.26, 0.13–1.31)	0.43 (0.15, 0.12–0.75)	4/5	0.196
Immigrant stress score (0–2)	0.85 (0.38, 0–1.75)	0.99 (0.38, 0.2–1.8)	2/3	**0.009**
Discrimination stress (0–2)	0.27 (0.37, 0–2)	0.21 (0.31, 0–1.4)	2/3	0.482
School-related stress (0–2)	0.51 (0.32, 0–1.43)	0.55 (0.28, 0–1.43)	3/4	0.126
Fear of parent's deportation			2/3	*0.085*
Never	27 (35.0%)	8 (22.2%)		
Sometimes	31 (40.3%)	19 (52.8%)		
Always	19 (24.7%)	9 (25%)		
**Child resilience factors**				
Social support parents (0–2)	1.41 (0.41, 0.5–2.0)	1.53 (0.34, 0.6–2.0)	2/3	**0.002**
Optimism (YLOT; 12–48)	–	32.83 (7.08, 20–46)	39/3	–
**Mother report of psychosocial stressors**				
Total maternal stress (0–1)	0.30 (0.17, 0.02–0.78)	0.25 (0.12, 0.02–0.53)	8/5	**0.019**
Immigrant stress score (0–1)	0.46 (0.23, 0–0.9)	0.42 (0.21, 0–0.8)	8/1	**0.002**
Discrimination score (0–1)	0.43 (0.25, 0–1.0)	0.36 (0.24, 0–0.86)	6/2	*0.053*
Household stress (0–1)	0.21 (0.21, 0.0–0.8)	0.23 (0.20, 0.0–0.8)	5/1	0.610
Family economic stress (0–1)	0.24 (0.24, 0.0–1.0)	0.17 (0.17, 0.0–0.63)	6/4	*0.059*
Health stress (0–1)	0.32 (0.26, 0.0–1.0)	0.27 (0.22, 0.0–1.0)	3/2	0.224
**Mother resilience variables**				
Subjective social status (1–10)	4.07 (1.88, 1–9)	4.32 (1.56, 1–8)	3/1	*0.075*
Social support and connection (1–4)	2.48 (0.58, 0.25–3.40)	2.19 (0.56, 1.0–3.40)	3/1	**0.019**
Optimism (0–24)	17.12 (3.05, 12–24)	13.66 (2.30, 8–18)	5/1	** <0.001**

† *P-value based on paired t-tests for continuous variables, and McNemar (paired Chi Square) test for categorical variables. Comparisons were calculated only on the 36 individuals in both time points. Bolded values represent significant associations. Italicized values represent marginal associations. Values in parentheses following psychosocial stressors and resilience factors represent the potential range of each measure*.

#### Stress and Resilience Levels

All measured stress and resilience levels at both time points are reported in Table 1. Generally, we found stress levels in mothers were high across both time points, with slight decreases in total stress and immigrant stress over time, while resilience factors such as optimism and social support also decreased over time. In children, stress and resilience measures both generally increased over time, particularly immigrant stress and social support from parents. There was wide variation across most of these measures in this sample, as reported previously (53).

### Correlations Among All Study Variables

Bivariate correlations between all study variables can be found in Supplementary Tables 3.0, 3.1, 4.0, 4.1. In brief, many correlations were found in expected directions between stress and resilience exposures with cardiometabolic risk factors and DNA methylation in both mothers and children, though results varied somewhat across generations and across time points. These exploratory analyses guided the building of linear models, which showed largely similar results, as described below.

### Associations Between Stressors and Cardiometabolic Markers

In linear models adjusted for key covariates at baseline, no variables associated with children's cardiometabolic markers, but in mothers, social support and connection inversely associated with BMI [Beta: −2.15 (SD <0.01), *p* = 0.034] (Table 2). At follow-up, children's school stress [B: 22.15 (10.72), *p* = 0.048] and everyday discrimination [B: 9.92 (3.72), *p* = 0.015] were positively associated with children's waist circumference. In the mothers at follow-up, a negative association was detected between acculturation (SASH) and BMI [B= −2.96 (1.44), *p* = 0.049], and between subjective social status and SBP [B: −2.71 (1.27), *p* = 0.040] (Table 2). Other marginal associations are displayed in Table 2.

**Table 2:** Children's and Mothers' psychosocial stressors, resilience factors, and cardiometabolic biomarkers.

		**Children's cardiometabolic health biomarkers**		
		**BMI percentile B (SD)**	**Waist circumferenceB (SD)**	
**Children's measures at follow-up**		*n* = 31	*n* = 30	
**Stress variables**				
	Total child stress score	–	*42.13 (21.09)*	
	School stress	–	**22.15 (10.72)**	
	Everyday discrimination	–	**9.92 (3.72)**	
	Discrimination stress	–	*20.16 (10.88)*	
		**Mother's cardiometabolic health biomarkers**		
		**BMI** **B (SD)**	**SBPB (SD)**	**DBP** **B (SD)**
**Mothers' measures at baseline**		*n* = 76	*n* = 73	*n* = 73
**Stress factors:**				
	Immigrant related stress	–	*12.04 (6.57)*	–
	Discrimination stress	–	*10.92 (6.29)*	–
**Resilience factors:**				
	Social support/social connection	**−2.15 (<0.01)**	–	–
	Optimism	*−0.34 (0.20)*	–	*0.59 (0.35)*
**Mothers' measures at follow-up**		*n* = 30	*n* = 34	*n* = 34
**Stress factors:**				
	Discrimination stress	–	*15.8 (8.37)*	–
**Resilience factors:**				
	Subjective Social Status	–	**−2.71 (1.27)**	–
	Social support/social connection	–	–	*5.11 (2.87)*
	Acculturation (SASH)	**−2.96 (1.44)**	–	–

*B, Beta, represents unstandardized regression coefficients; SD, standard deviation. Only significant (bolded) or marginalized (italicized) findings are presented*.

### Associations Between Stress/Resilience and *FKBP5* Methylation

At baseline in children, only the variables of fear of parent's deportation and everyday discrimination were marginally associated with *FKBP5* methylation (Table 3). In mothers at baseline, a significant negative association was found between mother's acculturation (SASH) and mother's *FKBP5* methylation, such that those who communicated more comfortably in English had lower methylation at the first CpG site [B: −1.19 (0.44), *p* = 0.008] and lower average methylation across sites of *FKBP5* [B: −0.89 (0.36), *p* = 0.017; Table 3].

**Table 3:** Psychosocial stressors and resilience factors and methylation levels at *FKBP5* of children and mothers at both time points.

	***FKBP5***		
**Baseline**	**CpG1**	**CpG2**	**Average**
**Child stress variables**	**B (SD)**	**B (SD)**	**B (SD)**
Fear of parent's deportation	–	*0.72 (0.37)*	–
Discrimination stress	*−1.93 (1.06)*	–	–
**Maternal resilience variables**			
Acculturation (SASH)	**−1.19 (0.44)**	*−0.58 (0.33)*	**−0.89 (0.36)**
**Follow-up**	**CpG1**	**CpG2**	**Average**
**Child stress variables**			
Everyday discrimination	–	**−1.03 (0.38)**	*−0.93 (0.45)*
**Child resilience variables:**			
Parental social support	**2.86 (1.29)**	–	*1.94 (1.07)*
**Maternal stress variables:**			
Family health stress	–	–	*−1.25 (0.64)*

*B, Beta, represents unstandardized regression coefficients; SD, standard deviation. Only significant (bolded) or marginalized (italicized) findings are presented*.

At follow-up, a negative association was found between child's report of Everyday Discrimination and children's methylation at the second site of *FKBP5* [B: −1.03 (0.38), *p* = 0.0141]. Child's report of parental social support was positively associated with methylation at the first site [B: 2.86 (1.29), *p* = 0.035] of *FKBP5* (Table 3).

### Associations Between Stress/Resilience and *SLC6A4* Methylation

In children at baseline, positive associations were detected between IRS with two sites in *SLC6A4*: CpG3 and CpG5 (Table 4). Children's fear of parent's deportation was also associated with CpG sites 3, 5, and 6 of *SLC6A4* (all *p* < 0.040). In mothers at baseline, various sites of *SLC6A4* were positively associated with total maternal stress, IRS, health stress, and discrimination stress (Table 4, all *p* < 0.040). SSS was negatively associated with CpG1, CpG3, CpG6, and average methylation (all *p* < 0.030).

**Table 4:** Psychosocial stressors and resilience factors and methylation levels at *SLC6A4* of children and mothers at both time points.

	***SLC6A4***						
	**CpG1**	**CpG2**	**CpG3**	**CpG4**	**CpG5**	**CpG6**	**Average**
**Baseline**							
**Child variables**	**B** **(SD)**	**B** **(SD)**	**B** **(SD)**	**B** **(SD)**	**B** **(SD)**	**B** **(SD)**	**B** **(SD)**
**Stress factors:**							
Total stress score	–	–	–	–	*0.98 (0.67)*	*1.34 (0.68)*	–
School stress	–	–	–	*0.73 (0.47)*	*0.78 (0.53)*	–	–
Immigrant-related stress	–	–	**0.77 (0.35)**	–	**0.89 (0.44)**	*0.89 (0.45)*	–
Fear of parent's deportation	–	–	**0.38 (0.17)**	*0.34 (0.19)*	**0.55 (0.21)**	**0.49 (0.21)**	*0.25 (0.15)*
**Maternal variables**							
**Stress factors:**							
Total stress score	*2.72 (1.37)*	1.85 (1.19)	*2.17 (1.20)*	–	–	**2.10 (0.93)**	**1.50 (0.70)**
Immigrant-related stress	**2.74 (0.98)**	**1.83 (0.85)**	**2.11 (0.86)**	–	–	**1.65 (0.67)**	**1.56 (0.49)**
Family health stress	–	–	**1.92 (0.76)**	–	–	**1.50 (0.60)**	**0.94 (0.45)**
Discrimination stress	**1.98 (0.95)**	–	–	–	–	–	**1.01 (0.49)**
**Resilience factors:**							
Subjective social status	**−0.25 (0.12)**	–	**−0.23 (0.10)**	*−0.13 (0.08)*	−0.02 (0.06)	**−0.24 (0.08)**	**−0.17 (0.06)**
Acculturation (SASH)	*−0.88 (0.44)*	–	–	–	–	–	–
**Follow-up**							
**Child variables**							
**Resilience factors:**							
Parental social support	–	*−1.21 (0.65)*	–	–	–	–	–
Optimism	**–**	**−0.07 (0.03)**	–	–	–	–	–
**Maternal variables**							
**Stress factors:**							
Immigrant- related stress	–	–	–	**−3.37 (1.30)**	–	–	–
Household stress	**–**	–	–	**−2.64 (0.99)**	–	–	–
**Resilience factors:**							
Acculturation (SASH)	–	*−0.51 (0.25)*	**0.54 (0.23)**	**0.84 (0.27)**	**0.61 (0.29)**	–	**0.61 (0.22)**

*B, Beta, represents unstandardized regression coefficients; SD, standard deviation. Only significant (bolded) or marginalized (italicized) findings are presented*.

In children at follow-up, optimism was negatively associated with CpG2 in *SLC6A4* [B: −0.07 (0.03), *p* = 0.040] (Table 4). In mothers we see some associations in the opposite direction as seen at baseline, as both IRS and household stress *negatively* associated with CpG4 of *SLC6A4* (all *p* < 0.02), while SASH was *positively* associated with CpG3, CpG4, CpG5, and average methylation at *SLC6A4* (Table 4).

### Associations Between Methylation and Cardiometabolic Markers

In the children at baseline, we detected significant inverse associations at baseline between several sites and the average methylation of *SLC6A4* with both BMI percentile and waist circumference (Table 5). Associations were consistently in the negative direction in the smaller sample size at follow-up, though not significant (data not shown). In the mothers at follow-up, we detected positive associations between multiple sites of *SLC6A4* and DBP—but not with SBP or BMI (Table 5).

**Table 5:** Children's and mothers' SLC6A4 methylation levels and cardiometabolic biomarkers.

	**Child's BMI percentile at baseline**	***p*-value**	**Child's waist circumference at baseline**	***p*-value**	**Mother's DBP at follow-up**	***p*-value**
	***n*** **=** **69**		***n*** **=** **62**		***n*** **=** **32**	
	**B (SD)**		**B (SD)**		**B (SD)**	
***SLC6A4***						
Average	**−11.75 (3.63)**	**0.002**	**−4.63 (1.54)**	**0.004**	**4.10 (1.61)**	**0.016**
CpG 1	**−3.91 (1.73)**	**0.027**	**−2.00 (0.71)**	**0.007**	–	–
CpG 2	–	–	*−2.49 (1.39)*	*0.078*	**3.46 (1.49)**	**0.027**
CpG 3	*−6.01 (3.27)*	*0.070*	**−2.91 (1.34)**	**0.033**	**3.64 (1.62)**	**0.032**
CpG 4	*−5.84 (2.95)*	*0.052*	–	–	**3.55 (1.24)**	**0.007**
CpG 5	**−5.94 (2.60)**	**0.025**	**−2.24 (1.08)**	**0.042**	**3.28 (1.23)**	**0.012**
CpG 6	**−7.00 (2.52)**	**0.007**	*−2.12 (1.20)*	*0.082*	**2.52 (1.15)**	**0.036**

*B, Beta, represents unstandardized regression coefficients; SD, standard deviation. Only significant (bolded) or marginalized (italicized) findings are presented*.

### Secondary Analyses: Cross-Generational and Longitudinal Analyses

#### Comparing Methylation Across Generations: Mothers vs. Children

At the baseline timepoint, paired *t*-tests revealed no significant differences between mothers and children across sites of *FKBP5*, though mothers tended to show lower methylation than children at CpG1 and the average. Mother's DNA methylation was significantly higher relative to the child's methylation in *SLC6A4* average and at sites CpG1, CpG2, and CpG4, (all *p* < 0.005, Supplementary Figure 1.0). At the follow-up time point, the mother's methylation was significantly higher than the child's at *SLC6A4* CpG4 (*t* = 2.41, df = 34, *p* = 0.022; Supplementary Figure 1.1).

#### Longitudinal Analyses

Children showed a significant increase over time in *SLC6A4* methylation at CpG1 (*t* = 2.15, df = 34, *p* = 0.039). Mothers showed a significant decrease in *FKBP5* methylation at CpG2 site (*t* = −2.86, df = 23, *p* = 0.009) and average across sites (*t* = −2.14, df = 23, *p* = 0.0430) (Supplementary Figure 2.0). We found the same site of CpG1 in *SLC6A4* in children to show significant change over time in a linear mixed model, after adjusting for key demographic covariates, suggesting that the methylation change was independent of age (and age was not significantly associated). For maternal methylation, no significant changes over time were detected for *FKBP5* after adjusting for covariates.

## Discussion

This study is the first, to our knowledge, to demonstrate the impact of daily stressors of immigrant families (mothers and children) on cardiometabolic risk and DNA methylation of stress-related genes over time. In general, stressors were associated with increased adiposity, while protective factors, such as social support and higher subjective social status were negatively associated with adiposity and BP. Associations with epigenetic factors were generally in the expected direction in both mothers and children within each gene, such that increased levels of stress were associated with higher DNA methylation in *SLC6A4*, and greater levels of resilience factors were associated with lower methylation. While fewer associations were detected with *FKBP5*, generally, greater stress was associated with lower DNA methylation, and greater resilience with higher methylation. These trends were consistent with prior studies of these gene regions, where greater stress was associated with higher methylation in the promoter of *SLC6A4*, and lower methylation in intron 7 of *FKBP5* (37, 38).

Below we describe in detail the magnitude and directions of associations, how our results relate to similar studies and contribute to theories of embodiment across the life course, and the potential functional relevance of findings.

### Embodiment via Cardiometabolic Risk Measures

We found children's stressors, such as everyday discrimination and school stress, positively associated with larger waist circumferences. This trend is consistent with other studies in adults that have shown stressors such as everyday discrimination to be associated with increased waist circumference (14) and low socioeconomic status to be related to greater abdominal fat deposit (68). In one study of Australian children (mean age 11 years), racial discrimination was associated with increased BMI, waist circumference, and SBP (14). A meta-analysis has documented strong associations between increased maternal stress and greater obesity in children (69), though fewer studies have measured stress experienced directly by children. Our data suggest that increased risk for obesity may be influenced by stress experienced by children in early to mid-childhood, even earlier in life than usually studied. This finding suggests the importance for health care providers to consider monitoring children's psychosocial stressors as contributors to cardiometabolic risk early in life, in order to curb progression of largely preventable cardiometabolic diseases.

In the mothers, we found greater acculturation and resilience factors (e.g., higher subjective social status, higher social support), associated with lower cardiometabolic risk, but little evidence that stress associated with either BMI or BP. A substantial literature supports associations between social support and BP and BMI; e.g., social support has been associated with lower BP reactivity to laboratory stress in older adults (70) and was protective against intergenerational transmission of obesity in a study of Finns in mid-adulthood (71). Findings with acculturation are more mixed. A systematic review found inconsistent effects of acculturation on BMI, such that 3 studies were consistent with our finding of lower BMI with more acculturation (which were mainly among women), while 6 studies of mixed gender showed the opposite pattern (72). BMI can be influenced by many complex factors, including cultural norms for body image, food availability, physical activity norms, and loss of a “healthy migrant effect” over time, such that it is challenging to predict how acculturation may influence this trait. Further research is needed to determine how acculturation and other resilience factors can protect against elevated cardiometabolic risk, to reduce the risk of multiple preventable health outcomes, including hypertension, diabetes, stroke, and overall mortality (73).

HPA axis dysregulation may be a mediating factor between psychosocial stress and increased risk of cardiometabolic disease. Many studies have found chronic stress from various sources associates with BMI, waist circumference, and adiposity, potentially linked through elevated cortisol, which influences metabolic function as well as stress response (74–76). For this reason, we examined DNA methylation of *FKBP5*, a gene related to HPA axis functioning. Additionally, the serotonergic system, including methylation of *SLC6A4*, is typically associated with social and emotional reactivity to stress (77), which has direct implications for cardiovascular health (78). Both general trends and specific findings associated with both genes are discussed below.

### Embodiment via Epigenetics

In comparing overall findings across methylation analyses, generally we found more significant associations between stressors and resilience factors with DNA methylation among mothers than among children. Though we measured more factors in the mothers, many of the same stressors were significantly associated with methylation in maternal saliva but not in children. In fact, we found mothers had lower levels of DNA methylation in *FKBP5* and higher levels in *SLC6A4* as compared to their children. Decreased methylation at *FKBP5* and increased at *SLC6A4* has been associated with increased stress in this and prior studies. These patterns may suggest that mothers are affected more by the daily stressors they are experiencing than their children, or potentially that mothers have accumulated more epigenetic changes over their life course than children. This result is counter to the hypothesis that childhood is a more sensitive period of development, and may instead support a role for the weathering hypothesis, where effects accumulate over time (26). Alternatively, the mother's own childhood exposures, many prior to migration (which were not measured in this study), may have been more adverse than that experienced by their US-born children.

In comparing across time points, significant epigenetic associations were found at similar frequencies at baseline and follow-up time points. In fact, in many cases the specific findings were similar across time points (with the exception of *SLC6A4* data in the mothers at follow-up, where the sample size was the smallest). The consistency of associations with methylation over time was unsurprising, giving the relative similar reports of stress exposures over time, and the relatively short time period of 2–3 years between samples. This consistency speaks to the relative stability of methylation data over time but also potentially the ongoing challenges faced by immigrant families over the past few decades, and the fact that more recent period of anti-immigrant focused politics in the US may not be a uniquely important stressor experienced in this population.

Despite measuring more stress than resilience factors, we found a surprisingly high number of associations with resilience factors in both mothers and children, justifying further study of epigenetics of resilience. While resilience can sometimes be viewed as simply the inverse of stress (i.e., lack of stress), our findings suggest there may be unique benefits to social support and optimism. These same factors have shown to buffer the effects of adverse childhoods on adult health behaviors and health outcomes (79), and thus the epigenetic effects we detect may be potential mediators along this pathway.

When comparing findings across tested genes, overall *SLC6A4* methylation was associated with more exposure variables than *FKBP5*. Specifically, we found very little significant associations with *FKBP5* in our baseline sample. Only maternal acculturation was associated with lower methylation at this gene, in the expected direction for a stressor in *FKBP5*. Interestingly, we found acculturation was also associated with lower methylation in *SLC6A4*, though higher methylation is expected for stress in this gene. While acculturation can be a stressful process, it can also have health benefits. Our measure of acculturation was a simple measure of language use. More detailed acculturation measures in the future could help clarify what components of acculturation are acting as risk or resilience factors for health, and how they relate to these epigenetic markers.

At the follow-up time point, we found children's reports of everyday discrimination associated with lower methylation at *FKBP5*, and parental social support with higher methylation. These associations were significant even after adjusting for covariates, and were in the expected direction based on prior studies where stress associates with lower methylation at this gene (36, 80). Our findings expand this literature by investigating these associations for the first time in children. The lack of significant findings at this gene in mothers relative to children in our sample may support the hypothesis that there are timing specific effects that may vary by gene, as found in previous longitudinal studies (38).

We found many different stressors associated with increased methylation (i.e., the expected direction) at *SLC6A4* in both children and mothers (e.g., immigrant related stress, fear of parent's deportation) at baseline, while greater subjective social stress was associated with decreased methylation in mothers. To our knowledge, SSS has never been evaluated with epigenetic data before, but appears to be protective against stress-related epigenetic changes in our findings. At follow-up, optimism was associated with lower methylation in children at this gene. In our prior research (53), we found increased optimism to be associated with increased epigenetic age in this same sample of children, which was attributed to potentially beneficial faster child development (e.g., reaching developmental milestones more rapidly). In mothers at follow-up, the few findings (mostly at one CpG site in *SLC6A4*) were inconsistent with baseline trends, and also inconsistent with prior studies. These findings should be interpreted with caution as they could be potentially spurious associations related to the much smaller sample size at follow-up.

In regards to the functional relevance of these results, one noteworthy finding was that methylation at *SLC6A4* was significantly associated with *lower* BMI percentile and waist circumference in the children, but *greater* BMI and higher BP in the mothers, at the follow-up time point. Similar to our findings in the children, one prior study found greater methylation of a CpG site in the same region in *SLC6A4* in blood of adolescents was associated with lower measures of adiposity including BMI, skin fold thickness, and waist circumference (17). Their study also found lower methylation at the same site in obese compared with lean adults in adipose tissue. Another study found no association between methylation at this gene and BMI in adults (81). These findings suggest directions of effect may vary with age, but further study in larger longitudinal samples is needed to clarify these trends.

Given that these same sites of *SLC6A4* also associated with stress factors in our dataset, taken together with these cardiometabolic findings, our data suggest that methylation may be a pathway through which stressful experiences like discrimination can become embodied and ultimately affect cardiometabolic health. Larger and longer-term longitudinal studies will ultimately be needed to formally address the role of this gene in mediating cardiometabolic outcomes across the life course.

### Longitudinal Trends

Over time, we found a decrease in mothers' average methylation levels at *FKBP5* and an increase in children's methylation at one CpG site in *SLC6A4*. For *SLC6A4* specifically, we found the effect persisted after adjusting for age, suggesting the increased methylation was not just an aging effect. These longitudinal trends, though small in magnitude, are in the direction expected for increased stress in their environments. In contrast, our measures of stress did not increase over time in mothers, but they did in children, who may have become increasingly aware of adverse sociopolitical environments as they aged. While there have been increasing policies directed against Latinx people during this 2-year study period, these changes may not represent a unique moment in time for this population, who have consistently faced high levels of discrimination and anti-immigrant policies for decades. Thus, this current sociopolitical moment may be less impactful than a lifetime of exposure to these stressors experienced by the mothers, consistent with the weathering hypothesis.

### Limitations and Strengths

Several limitations should be noted. First, interpretation of our findings is limited by relatively small sample sizes, especially at the follow up time point. The large attrition was expected, considering how recruitment was limited by several factors regarding the socio-politically stressful time period. This is typical in studies of vulnerable populations as there are inherent difficulties in recruiting a largely undocumented immigrant population (82). The high level of attrition may have biased our sample to retain less vulnerable members of the baseline group, particularly as those who left were more likely to be undocumented and unmarried. This attrition bias could contribute to the lack of a significant increase in stress over the studied time period, despite increasing anti-immigrant policies and rhetoric throughout this period. A second limitation is the lack of generalizability of our findings. While Nashville, TN represents a growing site for immigration settlement, our study is only based in one location in the US South, and the majority of the families were from Mexico, low SES, undocumented. Third, we recognize BMI is not an ideal marker of metabolic risk, as it is biased by design to measure “normal weight” for Western Europeans (83). However, it often has clinical relevance, especially when paired with waist circumference. Fourth, any analysis of DNA methylation in saliva samples cannot be generalized to other tissues of interest. Because saliva samples are made up of both epithelial cells and blood cells, there are concerns of cellular heterogeneity, that are generally difficult to account for with bioinformatic adjustment (84), and is typically only possible in genome-wide studies. Future studies are needed to address this issue, ideally using samples of isolated cell types across tissues. We also note that the methylation detected by bisulfite conversion techniques can't distinguish between 5-hydroxymethylcytosine (5hmC) and 5-methylcytosine (5mC), and thus our methylation estimates represent a mix of both nucleotides (85). Fifth, we only investigated a limited number of sites within two genes; however, these sites were carefully chosen based on prior studies to represent known pathways of stress embodiment. Finally, we recognize that if a Bonferroni correction were applied, few of our associations would pass. However, we note that in an exploratory study of methylation, Bonferroni can be overly conservative, particularly given that methylation levels are correlated across sites, as are the stressors, so they should not all be considered independent tests.

Our study contains a number of unique strengths as well. First, despite a small sample size, our study was unique in the depth of collected data, which includes comprehensive measures of psychosocial stress and resilience. Our survey data were obtained through extensive 2–3-h interviews with the mothers and an hour with the children. Another strength lies in the depth of data from two perspectives—mothers and children. This is especially important as children's perspectives on stress are very underrepresented in the literature, and provide insights on different epigenetic dynamics in different life stages. Further, the longitudinal design permits comparison of stress and methylation across different sociopolitical periods and across a ~2-year span of childhood. Longitudinal methylation data are very rare, especially during a difficult sociopolitical period for this population. This study is also one of the only studies of stress and methylation in Latinos. Finally, our targeted hypothesis-driven epigenetic approach is valuable, particularly for a focused study with extensive and detailed sociocultural data that are usually very challenging to collect on larger samples necessitated by genome-wide approaches (29).

## Conclusion

Our findings demonstrate an epigenetic pathway through which early adversity and ongoing stressful life events associate with DNA methylation within important regulatory regions of two well-characterized stress-related genes. This study explored these epigenetic associations within the context of a shifting sociopolitical environment. Many different stressors were associated with both cardiometabolic health markers and DNA methylation in both children and mothers at both genes and across time points. Associations between methylation at *SLC6A4* with cardiometabolic markers implies potential functional relevance of methylation at these sites in contributing to obesity and other cardiovascular or metabolic diseases. Taken all together, our findings suggest that methylation may be a pathway through which stressful experiences like discrimination can become embodied and ultimately affect cardiometabolic health.

## Data Availability Statement

The original contributions presented in the study are included in the article/Supplementary Materials, further inquiries can be directed to the corresponding author.

## Ethics Statement

The studies involving human participants were reviewed and approved by Vanderbilt University IRB-Human Research Protections Program; University of California San Diego IRB-Human Research Protections Program. Written informed consent from the participants' legal guardian/next of kin was not required to participate in this study in accordance with the national legislation and the institutional requirements. Oral informed consent was obtained from all participants.

## Author Contributions

EC and AN conceived of the study. AN coordinated and oversaw all study activities. EC generated the methylation data. All authors contributed to the analysis of the results and wrote the manuscript.

## Conflict of Interest

The authors declare that the research was conducted in the absence of any commercial or financial relationships that could be construed as a potential conflict of interest.
