# Supplementary material for: Epigenetics as a Mechanism of Developmental Embodiment of Stress, Resilience, and Cardiometabolic Risk Across Generations of Latinx Immigrant Families
Source: Front Psychiatry. 2021 Jul 20;12:696827. doi: 10.3389/fpsyt.2021.696827 (PMC8329078; doi:10.3389/fpsyt.2021.696827)
Supplement: Supplementary file 1 [file Data_Sheet_1.docx]

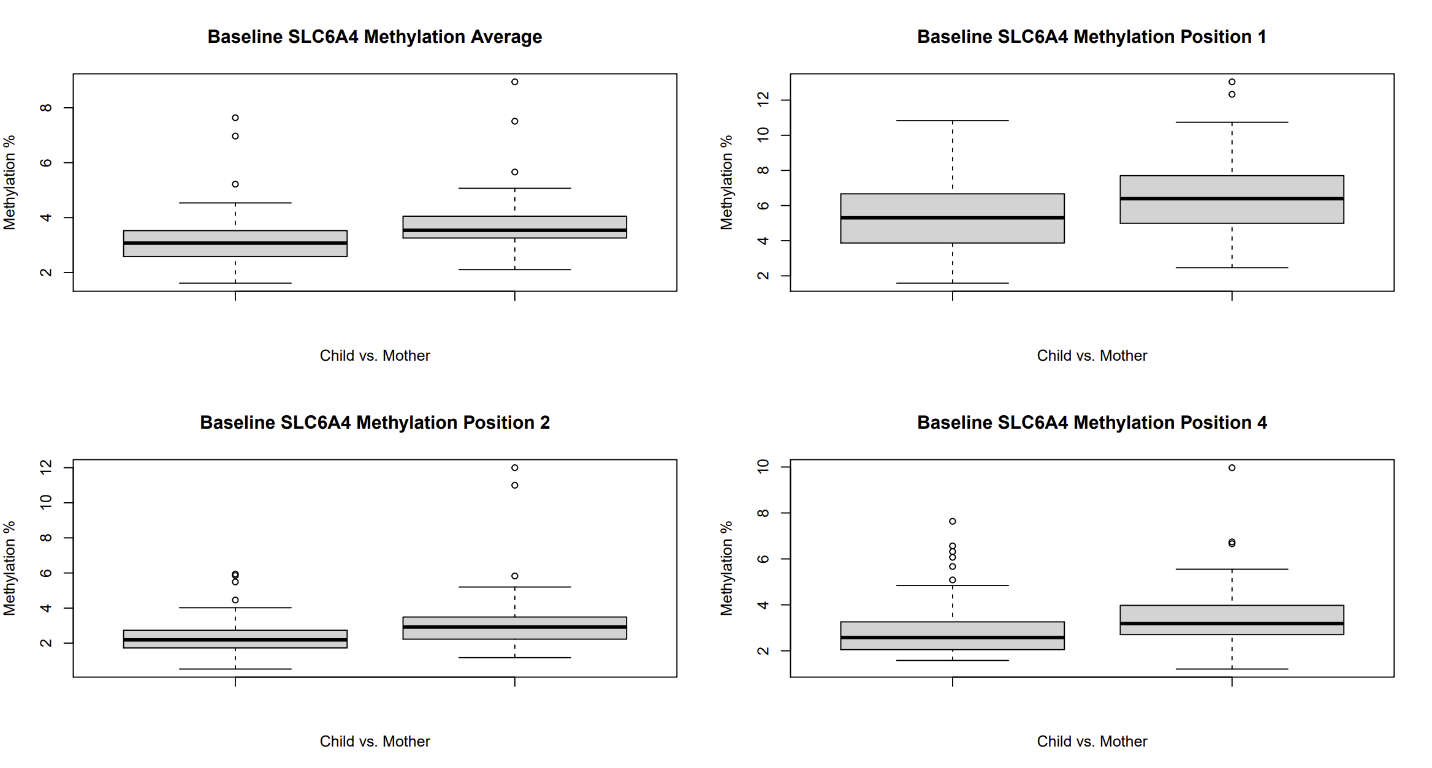


Supplemental Figure S1.0: Comparison of child and mothers’ methylation levels at *SLC6A4* sites showing significant differences at the baseline time point.


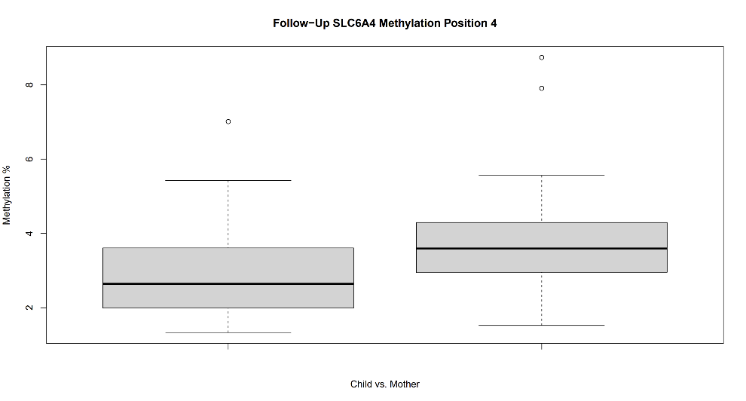


***SLC6A4* Methylation at CpG 4 at Follow-Up**

**Child Mother**

**Methylation %**

Supplemental Figure S1.1: Comparison of child and mothers’ methylation levels at CpG4 of *SLC6A4* at the follow-up time point.


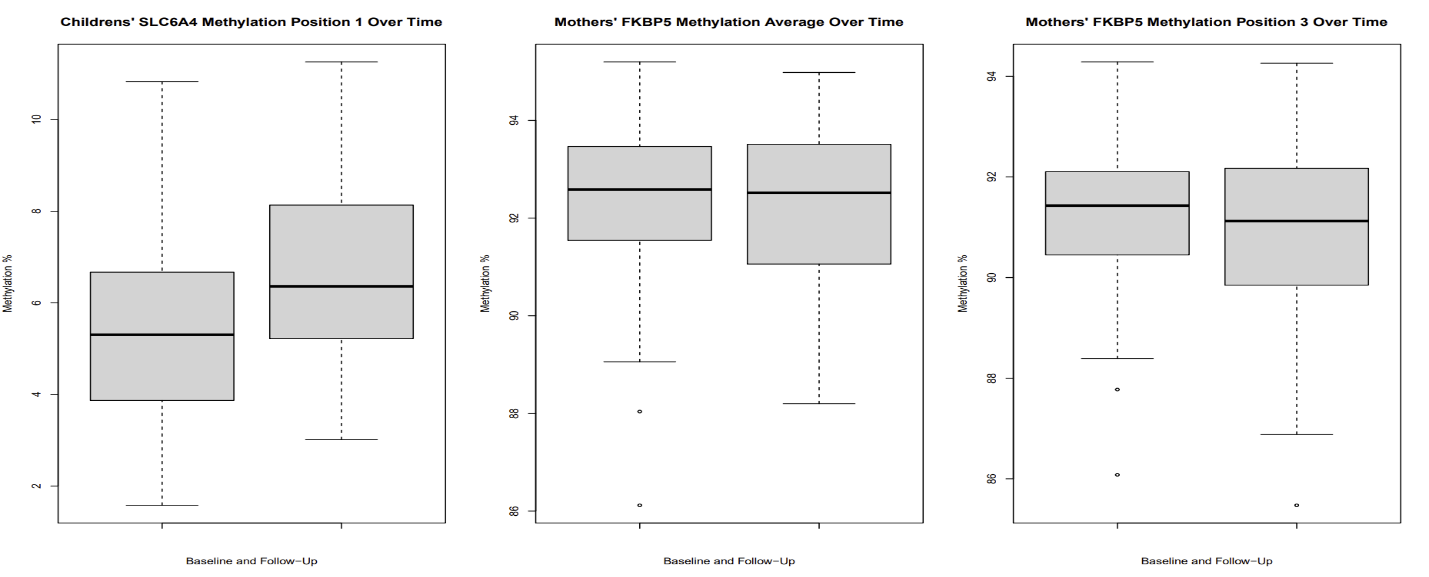


**Children’s *SLC6A4* Methylation Position 1 Over time**

**Mothers’ *FKBP5* Methylation Average Over time**

**Mothers’ *FKBP5* Methylation Position 2 Over time**

**Baseline Follow-Up**

**Baseline Follow-Up**

**Baseline Follow-Up**

A

B

C

Supplemental Figure S2.0: Mothers and children’s methylation levels over time (baseline and follow-up). A) presents children’s methylation at CpG1 of *SLC6A4*; B) presents mother’s average methylation levels across *FKBP5* sites, and C) presents mother’s methylation levels of CpG2 of *SLC6A4*.

Supplemental Table S1.0: Methylation values, age, child's gender, and cardiometabolic health biomarkers.

| Sample ID | Time Point | CpG1_*SLC6A4* | CpG2_ *SLC6A4* | CpG3_ *SLC6A4* | CpG4_ *SLC6A4* | CpG5_ *SLC6A4* | CpG6_ *SLC6A4* | Avg_ *SLC6A4* | CpG1_*FKBP5* | CpG2_ *FKBP5* | Avg_ *FKBP5* | Age (years) | Child’s Gender | BMI* | Systolic BP | Diastolic BP | Waist Circumference |
| --- | --- | --- | --- | --- | --- | --- | --- | --- | --- | --- | --- | --- | --- | --- | --- | --- | --- |
| CH_01 | BL | 6.67 | 2.18 | 2.96 | 2.93 | 1.34 | 3.27 | 3.22 | 91.76 | 88.76 | 90.26 | 8 | Girl | 97.80 | NA | NA | 56.75 |
| CH_02 | BL | 6.05 | 2.17 | 2.51 | 2.18 | 0.63 | 1.97 | 2.58 | 91.75 | 87.61 | 89.68 | 12 | Boy | 87.30 | NA | NA | 80.65 |
| CH_03 | BL | 8.28 | 3.71 | 3.31 | 2.35 | 0.76 | 3.03 | 3.57 | 91.07 | 89.10 | 90.08 | 7 | Boy | 74.50 | NA | NA | 62.60 |
| CH_04 | BL | 5.28 | 2.22 | 2.85 | 2.38 | 1.07 | 2.78 | 2.76 | 88.72 | 86.69 | 87.70 | 8 | Boy | 38.60 | NA | NA | 60.00 |
| CH_05 | BL | 7.15 | 2.17 | 1.91 | 3.24 | 0.68 | 6.00 | 3.53 | 94.54 | 89.66 | 92.10 | 8 | Boy | 48.40 | NA | NA | 61.80 |
| CH_07 | BL | 7.73 | 2.80 | 3.95 | 3.03 | 1.03 | 3.30 | 3.64 | 94.83 | 92.17 | 93.50 | 6 | Boy | 31.20 | NA | NA | 53.75 |
| CH_08 | BL | 10.83 | 2.37 | 2.77 | 2.33 | 0.76 | 3.17 | 3.70 | 91.54 | 91.71 | 91.63 | 8 | Girl | 54.80 | NA | NA | 57.50 |
| CH_09 | BL | 1.58 | 0.61 | 1.12 | 3.85 | 0.47 | 2.03 | 1.61 | 88.15 | 89.73 | 88.94 | 11 | Girl | 78.20 | NA | NA | 71.70 |
| CH_11 | BL | 7.57 | 3.77 | 3.73 | 3.50 | 1.22 | 3.53 | 3.88 | 94.87 | 91.32 | 93.09 | 6 | Girl | 86.40 | NA | NA | 57.50 |
| CH_12 | BL | 4.21 | 2.76 | 4.09 | 2.62 | 1.34 | 3.76 | 3.13 | 90.24 | 88.72 | 89.48 | 13 | Girl | 22.10 | NA | NA | 65.50 |
| CH_13 | BL | 5.66 | 2.74 | 2.88 | 2.41 | 0.68 | 3.31 | 2.94 | 92.71 | 90.90 | 91.81 | 8 | Girl | 29.10 | NA | NA | 59.50 |
| CH_14 | BL | 5.08 | 2.13 | 2.88 | 2.97 | 0.81 | 3.65 | 2.92 | 92.18 | 89.75 | 90.97 | 10 | Girl | 98.60 | NA | NA | NA |
| CH_15 | BL | 8.74 | 2.44 | 3.27 | 2.62 | 1.20 | 3.23 | 3.58 | 91.63 | 91.23 | 91.43 | 8 | Girl | 98.40 | NA | NA | NA |
| CH_16 | BL | 5.12 | 1.50 | 2.48 | 1.91 | 1.28 | 3.21 | 2.58 | 91.82 | 90.14 | 90.98 | 8 | Boy | 74.50 | NA | NA | 60.15 |
| CH_17 | BL | 8.63 | 3.25 | 2.94 | 6.32 | 1.34 | 4.04 | 4.42 | 91.33 | 90.80 | 91.06 | 11 | Boy | 19.60 | NA | NA | 71.75 |
| CH_18 | BL | 3.52 | 1.24 | 4.62 | 2.65 | 1.66 | 4.01 | 2.95 | 93.33 | 92.10 | 92.71 | 10 | Girl | 91.60 | NA | NA | 74.65 |
| CH_19 | BL | 6.05 | 2.65 | 3.46 | 2.38 | 0.88 | 3.15 | 3.09 | 93.67 | 90.56 | 92.11 | 7 | Boy | 98.80 | NA | NA | 73.00 |
| CH_20 | BL | 3.93 | 1.87 | 2.36 | 3.48 | 1.30 | 4.99 | 2.99 | 91.90 | 89.84 | 90.87 | 6 | Girl | 52.40 | NA | NA | 59.75 |
| CH_21 | BL | 7.88 | 2.19 | 3.34 | 2.36 | 1.62 | 3.53 | 3.48 | 95.60 | 92.78 | 94.19 | 7 | Girl | 3.90 | NA | NA | 50.55 |
| CH_22 | BL | 7.49 | 2.31 | 2.69 | 2.42 | 0.97 | 3.81 | 3.28 | 95.76 | 92.62 | 94.19 | 7 | Boy | 87.90 | NA | NA | 61.20 |
| CH_23 | BL | 6.46 | 1.94 | 3.55 | 2.64 | 1.31 | 3.24 | 3.19 | 94.51 | 91.00 | 92.75 | 9 | Boy | 13.60 | NA | NA | 67.95 |
| CH_25 | BL | 6.30 | 2.38 | 3.04 | 2.81 | 1.02 | 4.10 | 3.27 | 91.55 | 92.94 | 92.24 | 6 | Girl | 97.70 | NA | NA | 63.35 |
| CH_26 | BL | 7.32 | 5.94 | 7.58 | 7.64 | 6.55 | 6.80 | 6.97 | 96.26 | 93.72 | 94.99 | 7 | Girl | 25.50 | NA | NA | 58.75 |
| CH_27 | BL | 5.20 | 2.19 | 3.17 | 1.81 | 0.89 | 2.87 | 2.68 | 93.42 | 90.37 | 91.90 | 8 | Girl | NA | NA | NA | NA |
| CH_28 | BL | 5.76 | 1.26 | 2.37 | 1.99 | 0.70 | 2.55 | 2.44 | 95.23 | 88.51 | 91.87 | 7 | Boy | 86.30 | NA | NA | 63.30 |
| CH_29 | BL | 1.97 | 1.31 | 2.19 | 1.90 | 0.97 | 3.49 | 1.97 | 98.33 | 94.34 | 96.34 | 7 | Girl | 95.10 | NA | NA | 63.05 |
| CH_30 | BL | 5.46 | 2.50 | 3.65 | 2.34 | 1.23 | 5.55 | 3.45 | 92.29 | 91.24 | 91.76 | 10 | Girl | 98.60 | NA | NA | 95.00 |
| CH_31 | BL | 7.39 | 2.93 | 2.34 | 2.15 | 1.42 | 4.09 | 3.38 | 96.26 | 89.31 | 92.78 | 8 | Boy | 64.40 | NA | NA | 63.50 |
| CH_32 | BL | 5.93 | 4.46 | 2.93 | 3.68 | 1.96 | 2.32 | 3.55 | 93.45 | 93.25 | 93.35 | 7 | Boy | 89.10 | NA | NA | 64.15 |
| CH_33 | BL | 3.95 | 2.55 | 3.14 | 2.73 | 1.39 | 3.45 | 2.86 | 87.43 | 88.61 | 88.02 | 6 | Boy | 72.90 | NA | NA | 55.60 |
| CH_34 | BL | 5.44 | 1.72 | 3.09 | 2.56 | 1.02 | 3.40 | 2.87 | 88.11 | 86.81 | 87.46 | 8 | Girl | 97.50 | NA | NA | 83.10 |
| CH_35 | BL | 4.41 | 1.44 | 4.36 | 2.46 | 1.06 | 3.81 | 2.92 | 92.22 | 92.77 | 92.49 | 9 | Girl | 54.40 | NA | NA | 68.50 |
| CH_36 | BL | 6.01 | 2.60 | 2.70 | 2.03 | 1.55 | 3.72 | 3.10 | 93.63 | 90.03 | 91.83 | 9 | Girl | 96.00 | NA | NA | 74.30 |
| CH_37 | BL | 4.28 | 2.41 | 2.67 | 2.32 | 1.52 | 3.27 | 2.74 | 95.31 | 84.19 | 89.75 | 12 | Girl | 90.80 | NA | NA | 84.75 |
| CH_38 | BL | 5.96 | 5.49 | 8.36 | 6.57 | 10.28 | 9.17 | 7.64 | 97.77 | 94.90 | 96.33 | 12 | Girl | 13.30 | NA | NA | 59.65 |
| CH_39 | BL | 5.86 | 2.17 | 2.77 | 4.84 | 1.15 | 3.40 | 3.36 | 95.32 | 90.87 | 93.09 | 8 | Girl | 14.20 | NA | NA | 56.50 |
| CH_40 | BL | 2.86 | 2.53 | 3.39 | 4.17 | 2.16 | 4.92 | 3.34 | 96.13 | 92.17 | 94.15 | 7 | Boy | 99.10 | NA | NA | 76.80 |
| CH_41 | BL | 8.12 | 4.02 | 2.96 | 2.92 | 1.17 | 2.81 | 3.67 | 92.34 | 85.57 | 88.96 | 7 | Boy | 99.40 | NA | NA | 80.25 |
| CH_42 | BL | 1.91 | 1.55 | 2.23 | 2.00 | 1.07 | 4.05 | 2.13 | 81.86 | 87.84 | 84.85 | 11 | Girl | 99.30 | NA | NA | 103.30 |
| CH_43 | BL | 5.38 | 1.49 | 2.69 | 1.98 | 1.09 | 3.27 | 2.65 | 94.04 | 91.25 | 92.64 | 6 | Boy | 86.90 | NA | NA | 57.65 |
| CH_44 | BL | 7.57 | 1.91 | 2.33 | 2.14 | 0.74 | 2.55 | 2.87 | 97.25 | 93.02 | 95.13 | 11 | Boy | 96.60 | NA | NA | 94.40 |
| CH_45 | BL | 3.87 | 1.30 | 2.05 | 1.80 | 0.87 | 3.73 | 2.27 | 95.88 | 92.75 | 94.31 | 12 | Girl | NA | NA | NA | NA |
| CH_46 | BL | 3.59 | 2.08 | 3.51 | 1.66 | 0.82 | 2.33 | 2.33 | 93.06 | 91.62 | 92.34 | 11 | Girl | 74.90 | NA | NA | 79.13 |
| CH_47 | BL | NA | NA | NA | NA | NA | NA | NA | 85.22 | 94.91 | 90.07 | 5 | Boy | 99.90 | NA | NA | 73.67 |
| CH_48 | BL | 5.56 | 2.25 | 4.40 | 3.50 | 1.72 | 3.85 | 3.54 | 97.30 | 92.76 | 95.03 | 6 | Girl | 80.00 | NA | NA | 56.50 |
| CH_49 | BL | 4.46 | 1.77 | 3.31 | 1.83 | 1.77 | 2.79 | 2.65 | 92.42 | 93.00 | 92.71 | 8 | Boy | 90.30 | NA | NA | 66.55 |
| CH_50 | BL | 7.30 | 5.86 | 2.34 | 2.26 | 0.97 | 2.40 | 3.52 | 95.18 | 91.78 | 93.48 | 12 | Girl | 97.70 | NA | NA | 95.75 |
| CH_51 | BL | 3.74 | 3.21 | 4.33 | 5.67 | 2.69 | 4.50 | 4.02 | 95.17 | 93.66 | 94.41 | 11 | Girl | 93.90 | NA | NA | 76.33 |
| CH_52 | BL | 5.64 | 2.38 | 3.24 | 3.01 | 2.28 | 3.20 | 3.29 | 97.58 | 90.57 | 94.08 | 9 | Boy | 52.40 | NA | NA | 61.55 |
| CH_53 | BL | 4.51 | 0.90 | 2.21 | 1.78 | 0.86 | 2.78 | 2.17 | 94.00 | 93.07 | 93.53 | 7 | Boy | 40.10 | NA | NA | 44.30 |
| CH_54 | BL | 3.26 | 1.61 | 2.49 | 2.67 | 1.43 | 2.88 | 2.39 | 95.72 | 92.94 | 94.33 | 7 | Boy | 98.90 | NA | NA | 76.20 |
| CH_55 | BL | 3.36 | 0.53 | 2.20 | 3.79 | 0.44 | 4.86 | 2.53 | 98.25 | 94.71 | 96.48 | 7 | Boy | 99.90 | NA | NA | 105.73 |
| CH_56 | BL | 4.04 | 1.27 | 2.85 | 3.34 | 1.91 | 3.05 | 2.74 | 91.46 | 92.67 | 92.06 | 12 | Girl | 90.30 | NA | NA | 90.33 |
| CH_57 | BL | 5.27 | 1.50 | 3.74 | 2.76 | 0.95 | 3.31 | 2.92 | 95.15 | 91.56 | 93.35 | 10 | Girl | 95.70 | NA | NA | 80.55 |
| CH_58 | BL | 7.98 | 3.06 | 3.71 | 4.78 | 3.64 | 4.05 | 4.53 | 92.60 | 89.85 | 91.22 | 9 | Girl | 84.60 | NA | NA | NA |
| CH_59 | BL | 7.65 | 3.67 | 3.26 | 3.26 | 2.50 | 3.81 | 4.02 | 93.64 | 86.78 | 90.21 | 12 | Boy | 52.40 | NA | NA | 61.50 |
| CH_60 | BL | 7.21 | 1.82 | 3.15 | 1.91 | 0.43 | 3.09 | 2.93 | 99.11 | 95.04 | 97.07 | 12 | Girl | 86.20 | NA | NA | 74.75 |
| CH_61 | BL | 2.36 | 2.27 | 2.36 | 2.33 | 1.24 | 3.21 | 2.29 | NA | NA | NA | 6 | Boy | 85.80 | NA | NA | 64.40 |
| CH_62 | BL | 4.54 | 1.49 | 2.93 | 2.63 | 0.99 | 2.37 | 2.49 | 92.04 | 87.15 | 89.60 | 13 | Girl | 96.50 | NA | NA | 88.77 |
| CH_63 | BL | 3.88 | 2.92 | 5.03 | 2.42 | 2.23 | 3.95 | 3.40 | 96.68 | 92.13 | 94.40 | 7 | Boy | 97.60 | NA | NA | 69.60 |
| CH_64 | BL | 4.52 | 3.07 | 5.16 | 6.07 | 3.45 | 9.05 | 5.22 | 90.53 | 91.18 | 90.85 | 9 | Boy | 93.90 | NA | NA | NA |
| CH_65 | BL | 2.62 | 2.58 | 3.05 | 2.87 | 1.95 | 6.72 | 3.30 | 91.82 | 89.90 | 90.86 | 12 | Girl | 27.80 | NA | NA | 64.40 |
| CH_66 | BL | 3.38 | 1.86 | 2.35 | 1.58 | 1.46 | 3.20 | 2.30 | 91.66 | 88.11 | 89.88 | 12 | Boy | 94.80 | NA | NA | 89.90 |
| CH_67 | BL | 2.54 | 1.38 | 2.55 | 1.85 | 1.17 | 3.45 | 2.16 | 96.34 | 90.65 | 93.49 | 7 | Girl | 99.40 | NA | NA | 83.85 |
| CH_68 | BL | 7.98 | 3.28 | 3.13 | 3.66 | 2.53 | 4.44 | 4.17 | 96.60 | 93.31 | 94.96 | 9 | Girl | 71.20 | NA | NA | 62.37 |
| CH_69 | BL | 4.58 | 2.55 | 3.50 | 3.46 | 1.15 | 4.24 | 3.25 | 96.70 | 91.17 | 93.93 | 7 | Girl | 98.60 | NA | NA | 74.45 |
| CH_70 | BL | 5.33 | 1.99 | 3.11 | 2.93 | 1.33 | 3.63 | 3.05 | 97.15 | 93.12 | 95.13 | 8 | Girl | 14.50 | NA | NA | 53.35 |
| CH_71 | BL | 2.72 | 1.54 | 2.30 | 1.76 | 0.81 | 2.37 | 1.92 | 89.87 | 91.37 | 90.62 | 11 | Boy | 98.90 | NA | NA | 98.05 |
| CH_72 | BL | 5.33 | 2.19 | 3.00 | 1.98 | 1.91 | 4.93 | 3.22 | 91.23 | 91.57 | 91.40 | 9 | Boy | 98.40 | NA | NA | 84.75 |
| CH_73 | BL | 4.82 | 2.86 | 3.24 | 3.41 | 2.59 | 6.91 | 3.97 | 97.30 | 94.64 | 95.97 | 9 | Girl | 4.60 | NA | NA | 57.79 |
| CH_75 | BL | 2.00 | 1.52 | 1.95 | 5.09 | 1.65 | 2.93 | 2.52 | 98.35 | 91.51 | 94.93 | 9 | Boy | 98.90 | NA | NA | 92.70 |
| CH_77 | BL | 3.25 | 1.85 | 3.37 | 2.05 | 1.26 | 3.01 | 2.47 | 95.17 | 91.66 | 93.41 | 9 | Girl | 44.40 | NA | NA | NA |
| CH_78 | BL | 6.63 | 2.19 | 3.41 | 1.94 | 1.59 | 3.56 | 3.22 | 93.82 | 88.96 | 91.39 | 7 | Girl | 99.40 | NA | NA | NA |
| CH_79 | BL | 3.02 | 2.51 | 2.16 | 2.20 | 1.42 | 3.26 | 2.43 | 96.87 | 93.85 | 95.36 | NA | NA | NA | NA | NA | NA |
| CH_80 | BL | 9.92 | 1.73 | 2.25 | 1.62 | 2.38 | 5.18 | 3.85 | 94.88 | 90.45 | 92.66 | 6 | Girl | 26.40 | NA | NA | 56.55 |
| CH_81 | BL | 2.32 | 2.01 | 5.09 | 2.74 | 2.32 | 3.34 | 2.97 | 93.16 | 95.05 | 94.10 | 6 | Girl | 95.70 | NA | NA | 62.12 |
| CH_82 | BL | 6.58 | 3.39 | 4.26 | 2.60 | 1.27 | 6.01 | 4.02 | 96.27 | 92.38 | 94.32 | 12 | Girl | 56.00 | NA | NA | 64.00 |
| CH_83 | BL | 3.03 | 2.02 | 2.64 | 2.03 | 1.43 | 2.91 | 2.34 | 96.02 | 92.62 | 94.32 | 7 | Boy | 98.10 | NA | NA | 74.55 |
| CH_85 | BL | 4.81 | 2.19 | 2.60 | 2.21 | 1.76 | 3.59 | 2.86 | 96.75 | 93.99 | 95.37 | 7 | Boy | 82.40 | NA | NA | NA |
| CH_04 | FU | 9.89 | 2.30 | 2.96 | 2.65 | 1.12 | 2.94 | 3.64 | 92.21 | 89.44 | 90.82 | 11 | Boy | 46.40 | NA | NA | 24.70 |
| CH_07 | FU | 10.30 | 6.70 | 5.63 | 7.01 | 2.61 | 7.15 | 6.56 | 92.75 | 90.35 | 91.55 | 9 | Boy | 59.10 | NA | NA | 60.50 |
| CH_08 | FU | 6.16 | 3.18 | 4.50 | 3.00 | 1.98 | 4.05 | 3.81 | 93.81 | 90.67 | 92.24 | 11 | Girl | 69.50 | NA | NA | 61.00 |
| CH_09 | FU | 8.07 | 2.64 | 2.97 | 5.12 | 3.87 | 5.01 | 4.61 | 96.97 | 90.97 | 93.97 | 10 | Girl | 87.50 | NA | NA | 69.67 |
| CH_10 | FU | 8.14 | 1.98 | 4.96 | 4.12 | 2.19 | 4.37 | 4.29 | 94.43 | 92.27 | 93.35 |  | Boy | 95.50 | NA | NA | 71.60 |
| CH_11 | FU | 8.48 | 5.15 | 2.69 | 2.63 | 1.33 | 3.57 | 3.97 | 95.87 | 88.09 | 91.98 | 9 | Girl | 68.80 | NA | NA | 62.15 |
| CH_12 | FU | 6.54 | 4.46 | 4.32 | 3.61 | 3.60 | 5.67 | 4.70 | 95.55 | 93.23 | 94.39 | 15 | Girl | 44.80 | NA | NA | 71.83 |
| CH_13 | FU | 7.92 | 3.02 | 3.46 | 2.95 | 1.85 | 3.85 | 3.84 | 93.09 | 89.79 | 91.44 | 11 | Girl | 29.10 | NA | NA | 61.50 |
| CH_14 | FU | 5.93 | 3.58 | 4.23 | 3.67 | 2.63 | 4.48 | 4.09 | 92.43 | 89.78 | 91.10 | 13 | Girl | 95.80 | NA | NA | 91.25 |
| CH_15 | FU | 8.37 | 6.15 | 7.08 | 5.43 | 2.75 | 5.91 | 5.95 | 91.99 | 91.71 | 91.85 | 10 | Girl | 96.20 | NA | NA | 30.65 |
| CH_16 | FU | 9.15 | 2.99 | 4.35 | 3.79 | 2.05 | 3.99 | 4.39 | 94.47 | 92.93 | 93.70 | 11 | Boy | 80.20 | NA | NA | 66.30 |
| CH_19 | FU | 7.83 | 2.59 | 4.61 | 2.01 | 1.23 | 4.75 | 3.84 | 94.09 | 90.96 | 92.53 | 9 | Boy | 99.00 | NA | NA | 34.60 |
| CH_21 | FU | 3.52 | 2.40 | 2.10 | 2.10 | 0.64 | 2.11 | 2.14 | 97.20 | 92.11 | 94.65 | 9 | Girl | 5.90 | NA | NA | 51.60 |
| CH_22 | FU | 10.05 | 4.73 | 3.99 | 3.43 | 2.59 | 2.66 | 4.57 | 96.23 | 94.58 | 95.40 | 9 | Boy | 90.70 | NA | NA | 68.00 |
| CH_26 | FU | 6.76 | 2.69 | 2.42 | 1.34 | 0.82 | 2.45 | 2.74 | 94.40 | 91.98 | 93.19 | 9 | Girl | 84.10 | NA | NA | 72.00 |
| CH_28 | FU | 7.82 | 1.86 | 5.05 | 2.99 | 1.67 | 3.74 | 3.85 | 91.30 | 89.64 | 90.47 | 10 | Boy | 97.40 | NA | NA | 79.50 |
| CH_31 | FU | 8.96 | 2.17 | 2.74 | 1.46 | 1.22 | 4.94 | 3.58 | 93.23 | 89.70 | 91.46 | 10 | Boy | 70.50 | NA | NA | 63.50 |
| CH_32 | FU | 3.57 | 2.02 | 1.99 | 1.65 | 0.44 | 3.02 | 2.11 | 92.13 | 92.48 | 92.30 | 10 | Boy | 96.90 | NA | NA | 79.00 |
| CH_33 | FU | 5.85 | 4.36 | 7.47 | 4.94 | 4.29 | 6.35 | 5.54 | 93.97 | 93.28 | 93.62 | 8 | Boy | 58.70 | NA | NA | 62.00 |
| CH_44 | FU | 10.17 | 4.06 | 1.75 | 4.94 | 4.42 | 1.95 | 4.55 | 93.89 | 93.03 | 93.46 | 13 | Boy | 94.40 | NA | NA | NA |
| CH_47 | FU | 11.26 | 3.73 | 4.62 | 4.49 | 1.98 | 1.69 | 4.63 | 96.97 | 93.69 | 95.33 | 8 | Boy | 99.50 | NA | NA | 88.90 |
| CH_50 | FU | 4.36 | 1.24 | 2.75 | 1.34 | 0.93 | 3.05 | 2.27 | 80.56 | 82.03 | 81.29 | 15 | Girl | 95.80 | NA | NA | 86.50 |
| CH_55 | FU | 3.02 | 2.61 | 3.00 | 2.64 | 0.95 | 3.52 | 2.62 | 96.06 | 91.78 | 93.92 | 8 | Boy | 96.10 | NA | NA | 67.70 |
| CH_56 | FU | 5.29 | 1.47 | 2.55 | 1.51 | 0.97 | 3.03 | 2.47 | 89.33 | 88.23 | 88.78 | 15 | Girl | 88.50 | NA | NA | NA |
| CH_58 | FU | 4.55 | 2.30 | 2.88 | 2.84 | 1.29 | 5.97 | 3.30 | 92.02 | 90.53 | 91.27 | 11 | Girl | 90.10 | NA | NA | 71.50 |
| CH_61 | FU | 5.47 | 2.26 | 3.40 | 1.99 | 1.52 | 3.61 | 3.04 | 89.51 | 90.80 | 90.15 | 9 | Boy | 98.00 | NA | NA | 31.00 |
| CH_63 | FU | 7.42 | 2.41 | 2.66 | 2.04 | 0.58 | NA | 3.02 | NA | NA | NA | NA | Boy | NA | NA | NA | NA |
| CH_64 | FU | 5.80 | 3.28 | 3.10 | 2.62 | 2.06 | 3.63 | 3.41 | 92.74 | 91.65 | 92.20 | 11 | Boy | 16.60 | NA | NA | 80.00 |
| CH_67 | FU | 6.36 | 2.15 | 3.05 | 2.35 | 1.58 | 3.66 | 3.19 | 92.77 | 91.50 | 92.13 | 10 | Girl | 99.40 | NA | NA | 96.00 |
| CH_68 | FU | 3.84 | 2.06 | 2.15 | 1.92 | 1.15 | 2.95 | 2.34 | 91.93 | 92.59 | 92.26 | 11 | Girl | 67.70 | NA | NA | 66.00 |
| CH_69 | FU | 5.22 | 1.74 | 1.90 | 2.27 | 1.63 | 4.79 | 2.92 | 95.10 | 91.17 | 93.13 | 10 | Girl | 98.30 | NA | NA | 84.00 |
| CH_70 | FU | 4.16 | 2.26 | 3.57 | 1.80 | 1.14 | 2.91 | 2.64 | 93.13 | 91.93 | 92.53 | 11 | Girl | 42.90 | NA | NA | 65.25 |
| CH_73 | FU | 3.99 | 1.76 | 2.32 | 1.66 | 0.99 | 3.49 | 2.37 | 91.98 | 90.51 | 91.24 | 11 | Girl | 32.60 | NA | NA | 26.00 |
| CH_75 | FU | 4.22 | 1.87 | 2.43 | 1.71 | 0.93 | 3.47 | 2.44 | 93.86 | 93.31 | 93.59 | 9 | Boy | 97.50 | NA | NA | 74.00 |
| CH_81 | FU | 6.86 | 2.21 | 3.24 | 2.73 | 1.02 | 1.72 | 2.96 | 93.66 | 91.78 | 92.72 | 6 | Girl | NA | NA | NA | 63.00 |
| CH_82 | FU | 6.36 | 2.21 | 2.28 | 2.67 | 1.51 | 3.44 | 3.07 | 93.96 | 90.76 | 92.36 | 15 | Girl | 64.10 | NA | NA | 72.00 |
| CH_83 | FU | 6.16 | 2.66 | 3.28 | 2.93 | 1.59 | 4.76 | 3.56 | 93.25 | 91.63 | 92.44 | 11 | Boy | NA | NA | NA | NA |
| MO_01 | BL | 7.29 | 3.08 | 4.13 | 3.55 | 2.18 | 4.31 | 4.09 | 93.21 | 90.58 | 91.89 | 37 | Girl | 33.36 | 109.00 | 74.00 | NA |
| MO_02 | BL | 8.11 | 3.10 | 3.85 | 3.90 | 0.53 | 4.07 | 3.92 | 96.20 | 92.97 | 94.58 | 37 | Boy | 30.13 | 113.33 | 76.00 | NA |
| MO_03 | BL | 5.68 | 3.76 | 5.98 | 5.16 | 0.88 | 5.23 | 4.45 | 93.53 | 91.60 | 92.56 | 25 | Boy | 26.50 | 101.67 | 66.33 | NA |
| MO_04 | BL | 6.65 | 2.72 | 2.90 | 3.10 | 1.27 | 4.05 | 3.45 | 93.73 | 90.61 | 92.17 | 38 | Boy | 28.78 | 119.50 | 79.50 | NA |
| MO_05 | BL | 7.41 | 2.94 | 2.96 | 3.16 | 1.60 | 3.63 | 3.61 | 93.80 | 92.11 | 92.95 | 30 | Boy | 30.45 | 106.33 | 77.67 | NA |
| MO_06 | BL | 5.87 | 3.47 | 4.18 | 5.33 | 2.85 | 5.39 | 4.51 | 92.97 | 91.21 | 92.09 | 37 | Girl | 36.43 | 105.67 | 63.33 | NA |
| MO_07 | BL | 7.32 | 3.72 | 4.18 | 4.14 | 3.10 | 5.38 | 4.64 | 92.04 | 90.45 | 91.24 | 43 | Boy | 28.53 | 111.00 | 68.50 | NA |
| MO_08 | BL | 8.35 | 4.81 | 5.45 | 4.02 | 2.62 | 5.15 | 5.07 | 94.28 | 91.43 | 92.85 | 28 | Girl | 34.72 | 106.00 | 73.50 | NA |
| MO_09 | BL | 6.18 | 3.54 | 4.45 | 2.97 | 2.26 | 4.97 | 4.06 | 94.69 | 91.43 | 93.06 | 36 | Girl | 40.46 | 128.00 | 82.00 | NA |
| MO_10 | BL | 9.32 | 5.20 | 4.38 | 5.50 | 3.58 | 1.69 | 4.94 | 95.80 | 91.78 | 93.79 | 37 | Boy | 24.42 | 101.67 | 69.67 | NA |
| MO_11 | BL | 6.76 | 2.54 | 2.67 | 2.79 | 0.57 | 4.25 | 3.26 | 95.68 | 92.59 | 94.13 | 33 | Girl | 39.48 | NA | NA | NA |
| MO_12 | BL | 6.72 | 3.59 | 3.19 | 3.78 | 2.23 | 4.15 | 3.94 | 91.33 | 89.98 | 90.65 | 31 | Girl | 25.02 | 115.00 | 71.67 | NA |
| MO_13 | BL | 3.43 | 3.03 | 3.85 | 3.00 | 1.48 | 3.97 | 3.13 | 90.57 | 89.80 | 90.18 | 37 | Girl | 30.06 | 106.67 | 69.67 | NA |
| MO_14 | BL | 7.82 | 2.41 | 3.81 | 3.97 | 1.03 | 4.06 | 3.85 | 94.90 | 91.86 | 93.38 | 29 | Girl | 28.60 | 101.50 | 71.50 | NA |
| MO_15 | BL | 7.22 | 1.62 | 3.45 | 4.68 | 2.38 | 3.51 | 3.81 | 93.34 | 91.43 | 92.38 | 39 | Girl | 28.54 | 106.33 | 77.67 | NA |
| MO_16 | BL | 2.81 | 2.91 | 3.75 | 3.99 | 2.62 | 4.79 | 3.48 | 96.58 | 93.64 | 95.11 | 28 | Boy | 34.58 | 145.67 | 93.00 | NA |
| MO_17 | BL | 7.75 | 4.23 | 5.28 | 5.55 | 6.07 | 5.09 | 5.66 | 93.21 | 90.60 | 91.90 | 39 | Boy | 35.01 | 131.33 | 75.33 | NA |
| MO_18 | BL | 4.26 | 1.84 | 5.34 | 4.21 | 3.84 | 3.72 | 3.87 | 94.45 | 90.24 | 92.34 | 45 | Girl | 22.88 | 95.00 | 62.50 | NA |
| MO_19 | BL | 4.52 | 2.38 | 4.10 | 3.79 | 1.62 | 3.42 | 3.30 | 95.97 | 91.48 | 93.72 | 34 | Boy | 45.05 | 93.50 | 76.00 | NA |
| MO_20 | BL | 13.04 | 11.00 | 12.84 | 2.54 | 1.81 | 12.43 | 8.94 | NA | NA | NA | 23 | Girl | 31.87 | 120.33 | 77.67 | NA |
| MO_21 | BL | 10.74 | 4.57 | 3.53 | 2.99 | 1.86 | 4.68 | 4.73 | 95.42 | 91.42 | 93.42 | 30 | Girl | 30.27 | 101.50 | 69.00 | NA |
| MO_22 | BL | 6.77 | 2.55 | 3.22 | 1.67 | 1.20 | 5.03 | 3.40 | 96.85 | 92.39 | 94.62 | 35 | Boy | 28.56 | NA | NA | NA |
| MO_23 | BL | 8.92 | 2.25 | 3.21 | 2.98 | 1.22 | 3.46 | 3.67 | 93.63 | 92.00 | 92.81 | 27 | Boy | 27.83 | 139.67 | 92.00 | NA |
| MO_24 | BL | 5.60 | 3.37 | 5.18 | 2.18 | 0.43 | 5.24 | 3.66 | 96.26 | 94.14 | 95.20 | 40 | Girl | 32.92 | 118.00 | 79.33 | NA |
| MO_25 | BL | 3.27 | 3.03 | 4.20 | 2.94 | 1.38 | 3.94 | 3.13 | 94.34 | 93.14 | 93.74 | 27 | Girl | 22.70 | 99.33 | 70.33 | NA |
| MO_26 | BL | 6.51 | 4.40 | 2.14 | 3.95 | 0.22 | 3.63 | 3.47 | 95.75 | 93.74 | 94.74 | 26 | Girl | 38.07 | 131.67 | 91.00 | NA |
| MO_27 | BL | 4.39 | 1.60 | 2.98 | 2.35 | 1.15 | 3.95 | 2.73 | 93.23 | 91.58 | 92.40 | NA | Girl | NA | NA | NA | NA |
| MO_28 | BL | 6.02 | 2.13 | 2.48 | 2.52 | 1.84 | 4.26 | 3.21 | 95.51 | 94.03 | 94.77 | 33 | Boy | 27.06 | 98.00 | 74.00 | NA |
| MO_29 | BL | 2.46 | 2.00 | 2.34 | 6.66 | 2.56 | 3.52 | 3.26 | 92.05 | 88.46 | 90.25 | 33 | Girl | 40.31 | 106.67 | 77.00 | NA |
| MO_30 | BL | 6.47 | 3.32 | 3.82 | 6.74 | 1.69 | 4.24 | 4.38 | 94.14 | 93.12 | 93.63 | 41 | Girl | 23.88 | 101.33 | 62.00 | NA |
| MO_31 | BL | 7.25 | 1.88 | 2.05 | 5.12 | 1.17 | 4.33 | 3.63 | 91.21 | 91.14 | 91.17 | 36 | Boy | 25.17 | 102.50 | 66.50 | NA |
| MO_32 | BL | 9.57 | 1.73 | 1.98 | 2.77 | 1.22 | 2.69 | 3.33 | 93.84 | 90.38 | 92.11 | 32 | Boy | 24.52 | 113.33 | 71.00 | NA |
| MO_33 | BL | 6.31 | 2.64 | 2.50 | 3.20 | 2.15 | 3.43 | 3.37 | 95.09 | 94.29 | 94.69 | 37 | Boy | 32.23 | 105.33 | 66.00 | NA |
| MO_34 | BL | 5.29 | 1.19 | 2.65 | 2.83 | 1.34 | 3.56 | 2.81 | 93.56 | 90.45 | 92.01 | 27 | Girl | 33.83 | 98.67 | 71.33 | NA |
| MO_35 | BL | 5.70 | 2.15 | 3.67 | 3.48 | 1.45 | 3.15 | 3.26 | 91.48 | 88.51 | 89.99 | 34 | Girl | 31.54 | 111.00 | 86.00 | NA |
| MO_36 | BL | 8.62 | 3.48 | 2.29 | 2.86 | 0.95 | 3.12 | 3.55 | 95.23 | 91.84 | 93.53 | 38 | Girl | 25.86 | 130.67 | 111.00 | NA |
| MO_37 | BL | 9.34 | 4.63 | 2.76 | 3.32 | 1.18 | 3.46 | 4.11 | 96.30 | 92.67 | 94.48 | 37 | Girl | 33.02 | 109.00 | 73.00 | NA |
| MO_38 | BL | 5.96 | 4.02 | 6.10 | 4.49 | 1.92 | 3.63 | 4.35 | 93.84 | 92.58 | 93.21 | 32 | Girl | 27.49 | 114.00 | 73.50 | NA |
| MO_39 | BL | 4.38 | 3.56 | 4.12 | 2.99 | 2.82 | 3.42 | 3.55 | 94.02 | 91.88 | 92.95 | 30 | Girl | 23.75 | 98.33 | 63.33 | NA |
| MO_40 | BL | 12.33 | 2.93 | 2.81 | 3.94 | 2.21 | 4.01 | 4.70 | 93.29 | 91.57 | 92.43 | NA | Boy | NA | NA | NA | NA |
| MO_41 | BL | 4.10 | 1.18 | 3.89 | 2.98 | 0.40 | 2.97 | 2.59 | 90.73 | 90.35 | 90.54 | 35 | Boy | 40.56 | 123.67 | 85.67 | NA |
| MO_42 | BL | 4.41 | 2.82 | 3.52 | 2.54 | 2.09 | 3.10 | 3.08 | 95.40 | 93.71 | 94.56 | 35 | Girl | 27.61 | 105.50 | 79.00 | NA |
| MO_43 | BL | 6.01 | 3.25 | 3.84 | 2.50 | 1.95 | 3.70 | 3.54 | 93.12 | 91.50 | 92.31 | 40 | Boy | 35.67 | 113.00 | 72.00 | NA |
| MO_44 | BL | 6.90 | 3.50 | 2.62 | 4.67 | 1.59 | 3.31 | 3.76 | 93.05 | 90.66 | 91.85 | 39 | Boy | 33.81 | 105.50 | 67.50 | NA |
| MO_45 | BL | 5.71 | 2.93 | 4.64 | 4.43 | 1.64 | 3.72 | 3.84 | 92.22 | 89.81 | 91.01 | NA | Girl | NA | NA | NA | NA |
| MO_46 | BL | 8.13 | 3.37 | 4.84 | 2.71 | 2.21 | 5.24 | 4.41 | 92.14 | 90.37 | 91.25 | 30 | Girl | 39.76 | 105.33 | 72.00 | NA |
| MO_47 | BL | 7.76 | 3.02 | 3.48 | 2.64 | 1.07 | 3.26 | 3.54 | 96.38 | 92.05 | 94.21 | 38 | Boy | 27.48 | 98.33 | 73.67 | NA |
| MO_48 | BL | 6.00 | 2.22 | 2.55 | 2.39 | 1.87 | 3.71 | 3.12 | 94.67 | 90.98 | 92.83 | 25 | Girl | 25.75 | 104.00 | 61.50 | NA |
| MO_49 | BL | 7.66 | 3.75 | 2.32 | 2.25 | 1.12 | 3.08 | 3.36 | 92.09 | 90.81 | 91.45 | 45 | Boy | 25.33 | 109.00 | 85.50 | NA |
| MO_50 | BL | 5.21 | 2.90 | 3.52 | 3.33 | 1.51 | 3.77 | 3.37 | 92.59 | 90.55 | 91.57 | 35 | Girl | 23.74 | 127.33 | 82.67 | NA |
| MO_51 | BL | 6.04 | 2.35 | 3.31 | 3.78 | 1.51 | 2.69 | 3.28 | 95.38 | 91.18 | 93.28 | 39 | Girl | 31.63 | 113.33 | 69.00 | NA |
| MO_52 | BL | 7.59 | 2.79 | 3.07 | 2.92 | 1.61 | 4.23 | 3.70 | 93.20 | 91.87 | 92.54 | 38 | Boy | 27.41 | 98.00 | 59.50 | NA |
| MO_54 | BL | 4.96 | 2.84 | 4.48 | 4.13 | 2.12 | 4.72 | 3.87 | 96.32 | 91.83 | 94.07 | 48 | Boy | 30.96 | 100.00 | 63.33 | NA |
| MO_55 | BL | 6.49 | 3.31 | 3.00 | 3.21 | 1.76 | 3.31 | 3.51 | 93.91 | 90.91 | 92.41 | 37 | Boy | 21.51 | 81.67 | 57.67 | NA |
| MO_56 | BL | 8.04 | 2.21 | 6.74 | 4.14 | 1.97 | 4.27 | 4.56 | 91.69 | 91.66 | 91.68 | 37 | Girl | 31.49 | 121.00 | 81.00 | NA |
| MO_57 | BL | 4.98 | 2.93 | 2.65 | 2.93 | 1.70 | 4.44 | 3.27 | 88.30 | 87.78 | 88.04 | 35 | Girl | 32.95 | 109.67 | 68.33 | NA |
| MO_58 | BL | 5.61 | 2.14 | 2.86 | 3.88 | 1.38 | 3.12 | 3.16 | 93.39 | 92.31 | 92.85 | 29 | Girl | 26.92 | 108.00 | 78.00 | NA |
| MO_59 | BL | 5.00 | 3.45 | 3.56 | 3.55 | 2.05 | 3.48 | 3.51 | 92.57 | 89.79 | 91.18 | 28 | Boy | NA | NA | NA | NA |
| MO_60 | BL | 6.91 | 3.13 | 3.90 | 2.77 | 2.77 | 4.28 | 3.96 | 89.72 | 88.39 | 89.06 | 34 | Girl | 30.23 | 86.00 | 78.00 | NA |
| MO_61 | BL | 4.37 | 2.46 | 2.81 | 2.78 | 2.15 | 3.97 | 3.09 | 88.82 | 89.77 | 89.29 | 40 | Boy | 24.85 | 96.00 | 65.00 | NA |
| MO_62 | BL | 10.04 | 3.43 | 4.58 | 2.70 | 4.68 | 4.05 | 4.91 | 95.42 | 91.27 | 93.35 | 35 | Girl | 38.77 | 128.00 | 74.50 | NA |
| MO_63 | BL | 4.20 | 1.99 | 2.58 | 4.19 | 0.75 | 3.51 | 2.87 | 94.05 | 91.16 | 92.60 | 26 | Boy | 32.25 | 99.00 | 72.00 | NA |
| MO_64 | BL | 4.45 | 2.00 | 2.96 | 2.39 | 1.02 | 2.33 | 2.53 | 92.16 | 91.01 | 91.59 | 30 | Boy | 39.69 | 112.67 | 78.33 | NA |
| MO_65 | BL | 5.49 | 2.44 | 4.04 | 2.35 | 1.62 | 4.28 | 3.37 | 92.50 | 89.82 | 91.16 | 39 | Girl | 23.30 | 114.67 | 72.67 | NA |
| MO_66 | BL | 6.71 | 2.55 | 4.10 | 3.67 | 1.05 | 3.90 | 3.66 | 95.41 | 91.22 | 93.31 | 39 | Boy | 33.96 | 116.33 | 81.33 | NA |
| MO_67 | BL | 3.12 | 1.64 | 2.56 | 2.31 | 1.40 | 2.85 | 2.31 | 94.43 | 92.42 | 93.43 | 39 | Girl | 29.58 | 118.00 | 74.67 | NA |
| MO_68 | BL | 4.45 | 1.67 | 2.26 | 3.32 | 0.88 | 3.24 | 2.63 | 92.98 | 90.11 | 91.55 | 34 | Girl | 39.62 | 133.00 | 79.67 | NA |
| MO_69 | BL | 6.51 | 2.62 | 2.79 | 1.40 | 0.54 | 2.74 | 2.77 | 92.07 | 90.77 | 91.42 | 27 | Girl | 28.14 | 105.33 | 74.33 | NA |
| MO_70 | BL | 7.64 | 1.28 | 1.03 | 2.69 | 0.43 | 3.02 | 2.68 | 93.55 | 90.89 | 92.22 | 26 | Girl | 31.86 | 88.00 | 60.00 | NA |
| MO_71 | BL | 8.76 | 4.61 | 3.29 | 3.74 | 0.45 | 3.42 | 4.04 | 91.53 | 88.84 | 90.19 | 52 | Boy | 25.22 | NA | NA | NA |
| MO_72 | BL | 7.83 | 2.41 | 4.13 | 3.12 | 1.84 | 3.34 | 3.78 | 92.06 | 90.29 | 91.17 | 28 | Boy | 33.23 | 108.50 | 77.50 | NA |
| MO_75 | BL | 6.15 | 3.69 | 3.20 | 3.17 | 0.73 | 4.20 | 3.52 | 95.20 | 91.99 | 93.60 | 28 | Boy | 32.65 | 123.33 | 93.67 | NA |
| MO_77 | BL | 5.27 | 1.22 | 1.21 | 1.21 | 0.79 | 2.95 | 2.11 | 93.16 | 92.70 | 92.93 | 33 | Girl | 27.35 | 97.00 | 66.67 | NA |
| MO_78 | BL | 7.02 | 5.83 | 2.77 | 2.38 | 4.67 | 4.45 | 4.52 | 93.17 | 92.11 | 92.64 | 30 | Girl | 31.19 | 115.00 | 77.67 | NA |
| MO_79 | BL | 4.69 | 3.41 | 3.09 | 3.86 | 1.18 | 3.58 | 3.30 | 94.29 | 91.83 | 93.06 | NA | NA | NA | NA | NA | NA |
| MO_80 | BL | 7.80 | 4.27 | 2.31 | 4.87 | 1.37 | 4.57 | 4.20 | 95.21 | 92.22 | 93.71 | 45 | Girl | 23.34 | 92.00 | 60.33 | NA |
| MO_81 | BL | 5.08 | 1.73 | 3.32 | 3.84 | 1.38 | 3.15 | 3.08 | 95.18 | 91.76 | 93.47 | 44 | Girl | 22.57 | 99.67 | 68.67 | NA |
| MO_82 | BL | 3.06 | 12.00 | 10.53 | 9.97 | 1.22 | 8.28 | 7.51 | NA | NA | NA | 43 | Girl | 29.46 | 119.33 | 82.00 | NA |
| MO_83 | BL | 3.89 | 2.91 | 2.38 | 1.57 | 1.02 | 2.73 | 2.41 | 86.16 | 86.08 | 86.12 | 35 | Boy | 30.19 | 111.00 | 67.67 | NA |
| MO_85 | BL | 9.75 | 2.58 | 2.00 | 1.62 | 1.27 | 2.51 | 3.29 | 93.93 | 92.22 | 93.07 | 29 | Boy | 34.52 | 110.67 | 69.67 | NA |
| MO_04 | FU | 6.23 | 2.43 | 1.99 | 3.00 | 0.37 | 2.82 | 2.81 | 93.25 | 89.88 | 91.57 | 41 | Boy | 24.16 | 130.33 | 90.00 | NA |
| MO_06 | FU | 3.92 | 1.65 | 3.01 | 3.75 | 1.63 | 3.27 | 2.87 | 90.46 | 89.86 | 90.16 | 39 | Girl | 26.44 | 114.00 | 70.00 | NA |
| MO_07 | FU | 7.55 | 3.95 | 4.58 | 3.43 | 1.17 | 4.11 | 4.13 | 92.21 | 89.85 | 91.03 | 46 | Boy | 33.85 | 111.00 | 80.50 | NA |
| MO_08 | FU | 7.40 | 3.99 | 3.46 | 2.84 | 1.57 | 3.84 | 3.85 | 93.95 | 90.90 | 92.42 | 31 | Girl | 32.95 | 101.00 | 69.50 | NA |
| MO_09 | FU | 5.82 | 4.93 | 4.39 | 5.11 | 3.35 | 5.68 | 4.88 | 95.66 | 92.08 | 93.87 | 38 | Girl | 40.63 | 111.50 | 82.50 | NA |
| MO_10 | FU | 4.06 | 4.15 | 4.67 | 4.35 | 4.03 | 6.55 | 4.63 | 88.61 | 88.61 | 88.61 | 39 | Boy | 41.90 | 140.00 | 99.00 | NA |
| MO_11 | FU | 7.01 | 3.26 | 3.80 | 4.99 | 0.57 | 4.06 | 3.95 | 93.82 | 89.53 | 91.67 | 35 | Girl | 30.76 | 109.00 | 75.00 | NA |
| MO_12 | FU | 6.04 | 3.94 | 1.99 | 1.53 | 1.03 | 5.53 | 3.34 | 91.47 | 89.98 | 90.72 | 35 | Girl | 23.18 | 89.00 | 64.67 | NA |
| MO_13 | FU | 5.31 | 2.77 | 3.10 | 2.65 | 0.00 | 5.01 | 3.14 | 88.91 | 87.53 | 88.22 | 40 | Girl | 24.96 | 105.00 | 73.50 | NA |
| MO_14 | FU | 9.58 | 3.25 | 4.26 | 4.30 | 2.67 | 4.33 | 4.73 | 94.39 | 90.84 | 92.61 | 32 | Girl | 30.10 | 107.67 | 72.67 | NA |
| MO_15 | FU | 8.05 | 3.01 | 4.87 | 3.60 | 2.05 | 4.73 | 4.38 | 94.36 | 91.13 | 92.74 | 41 | Girl | 30.60 | 115.00 | 72.50 | NA |
| MO_16 | FU | 5.48 | 2.33 | 3.80 | 3.69 | 2.20 | 3.91 | 3.57 | 89.51 | 86.89 | 88.20 | 32 | Boy | 36.47 | 114.50 | 79.00 | NA |
| MO_19 | FU | 5.78 | 2.45 | 3.75 | 5.53 | 1.29 | 3.31 | 3.68 | 94.36 | 91.21 | 92.78 | 39 | Boy | 33.51 | 109.33 | 76.00 | NA |
| MO_21 | FU | 9.62 | 4.82 | 4.57 | 3.97 | 2.28 | 4.78 | 5.00 | 93.25 | 90.28 | 91.76 | 33 | Girl | 26.15 | 100.67 | 71.33 | NA |
| MO_22 | FU | 5.09 | 3.04 | 4.36 | 4.01 | 1.74 | 4.54 | 3.79 | 95.72 | 91.11 | 93.41 | 36 | Boy | 27.21 | 99.00 | 77.00 | NA |
| MO_24 | FU | 6.09 | 2.67 | 2.96 | 2.62 | 1.53 | 3.26 | 3.19 | 93.87 | 89.52 | 91.69 | 43 | Girl | 31.20 | 150.00 | 93.00 | NA |
| MO_26 | FU | 10.05 | 3.71 | 3.99 | 4.46 | 2.55 | 5.18 | 4.99 | 94.84 | 92.80 | 93.82 | 29 | Girl | NA | 120.00 | 87.00 | NA |
| MO_28 | FU | 7.11 | 1.72 | 1.79 | 1.83 | 1.51 | 2.03 | 2.66 | 93.65 | 92.07 | 92.86 | 36 | Boy | 33.09 | 100.00 | 69.00 | NA |
| MO_31 | FU | 5.28 | 2.51 | 2.70 | 3.32 | 1.52 | 3.68 | 3.17 | 94.86 | 92.23 | 93.54 | 37 | Boy | NA | 111.50 | 72.50 | NA |
| MO_32 | FU | NA | NA | NA | NA | NA | NA | NA | 92.98 | 91.63 | 92.30 | 34 | Boy | 29.56 | 115.00 | 72.50 | NA |
| MO_33 | FU | 6.68 | 2.70 | 3.21 | 3.34 | 1.98 | 4.18 | 3.68 | 92.76 | 91.12 | 91.94 | 39 | Boy | 34.28 | 129.00 | 78.50 | NA |
| MO_42 | FU | 5.61 | 1.92 | 2.53 | 2.94 | 1.38 | 3.59 | 2.99 | 94.69 | 92.34 | 93.51 | 38 | Girl | 40.09 | 124.50 | 74.00 | NA |
| MO_44 | FU | 8.44 | 3.73 | 3.97 | 5.21 | 1.74 | 3.48 | 4.42 | 94.10 | 91.52 | 92.81 | 41 | Boy | 23.67 | 110.50 | 75.50 | NA |
| MO_47 | FU | 6.97 | 3.16 | 2.82 | 3.50 | 1.89 | 3.96 | 3.71 | 95.33 | 92.80 | 94.06 | 40 | Boy | 31.83 | 124.00 | 78.00 | NA |
| MO_50 | FU | 5.77 | 2.52 | 3.44 | 2.96 | 4.98 | 4.80 | 4.08 | 88.01 | 89.25 | 88.63 | 38 | Girl | 36.96 | 117.00 | 85.00 | NA |
| MO_55 | FU | 7.78 | 4.63 | 3.78 | 3.72 | 1.42 | 3.75 | 4.18 | 93.77 | 93.20 | 93.48 | 40 | Boy | 39.95 | 110.00 | 88.50 | NA |
| MO_56 | FU | 6.89 | 3.04 | 3.06 | 3.80 | 2.74 | 2.99 | 3.75 | 93.17 | 90.73 | 91.95 | 40 | Girl | 30.76 | 110.00 | 75.00 | NA |
| MO_58 | FU | 7.39 | 2.51 | 3.00 | 3.14 | 1.79 | 5.05 | 3.81 | 93.70 | 91.87 | 92.78 | 32 | Girl | 25.53 | 103.00 | 70.50 | NA |
| MO_61 | FU | 8.02 | 5.04 | 5.35 | 5.56 | 4.90 | 8.18 | 6.17 | 93.12 | 88.96 | 91.04 | 43 | Boy | 25.32 | 113.00 | 71.00 | NA |
| MO_64 | FU | 6.77 | 7.14 | 6.63 | 8.74 | 5.67 | 8.70 | 7.27 | 93.45 | 89.48 | 91.46 | 32 | Boy | 18.67 | 128.00 | 112.50 | NA |
| MO_67 | FU | 3.19 | 0.75 | 2.23 | 2.19 | 1.29 | 3.05 | 2.12 | 95.21 | 94.26 | 94.74 | 44 | Girl | 42.57 | 142.50 | 80.50 | NA |
| MO_69 | FU | 7.13 | 3.09 | 3.37 | 3.09 | 0.52 | 3.16 | 3.39 | 90.60 | 91.52 | 91.06 | 31 | Girl | NA | 108.50 | 69.50 | NA |
| MO_70 | FU | 6.85 | 2.52 | 2.65 | 2.28 | 0.64 | 2.66 | 2.93 | 95.85 | 93.22 | 94.54 | 29 | Girl | 24.28 | 102.67 | 68.33 | NA |
| MO_73 | FU | 6.94 | 2.37 | 2.68 | 2.99 | 1.04 | 3.38 | 3.23 | 96.27 | 93.20 | 94.73 | 39 | Girl | 30.56 | 112.67 | 91.00 | NA |
| MO_75 | FU | 11.85 | 5.80 | 6.31 | 7.91 | 4.45 | 6.70 | 7.17 | 94.66 | 92.02 | 93.34 | 30 | Boy | 42.40 | 110.50 | 91.50 | NA |
| MO_81 | FU | 6.16 | 2.45 | 3.14 | 3.96 | 1.76 | 3.23 | 3.45 | 95.26 | 92.17 | 93.72 | 47 | Girl | NA | 116.00 | 78.50 | NA |
| MO_82 | FU | 10.52 | 2.90 | 2.96 | 2.80 | 1.08 | 3.10 | 3.89 | 96.46 | 93.51 | 94.98 | 46 | Girl | 31.41 | 129.50 | 81.50 | NA |
| MO_83 | FU | 5.07 | 3.13 | 3.57 | 3.84 | 2.45 | 4.44 | 3.75 | 91.39 | 85.48 | 88.43 | 38 | Boy | 34.55 | 120.00 | 80.00 | NA |

*In children, BMI percentile is adjusted for child’s age and gender. BL=baseline; FU=Followup; BP=blood pressure; NA=not available.

Supplemental Table S2.0. Targeted regions of *FKBP5* and *SLC6A4* for bisulfite pyrosequencing.

| **Gene** | **Chromosome location**  **(UCSC build hg19)** | **Amplicon** | **Primer Type** | **Primer sequence (5’🡪3’)** |
| --- | --- | --- | --- | --- |
| *FKBP5* | chr6:35,558,488-35,558,514 | 342 bp | Forward | EpigenDx ADS3828 FS2 (proprietary) |
|  |  |  | Reverse | EpigenDx ADS3828 FS2 (proprietary) |
|  |  |  | Sequencing | 5’ TGGAGTTATAGTGTAGGTTTT 3’ |
|  |  |  | Unconverted Sequence to analyze | 5’TTCGTGACTCCTGTGAAGGGTACAAT  1  CCGTTCA 3’  2 |
|  |  |  | Converted Sequence to analyze | 5’TTCGTGATTTTTGTGAAGGGTATAAT  1  TCGTTTA 3’  2 |
| *SLC6A4* | chr17:28,563,022-28,563,224 | 203 bp | Forward | 5’GTATTGTTAGGTTTTAGGAAGAAAGAGAGA 3’ |
|  |  |  | Reverse | 5’ Biotin- AAAAATCCTAACTTTCCTACTCTTTAACTT 3’ |
|  |  |  | Sequencing | 5’ AAGAAAGAGAGAGTAGTT 3’ |
|  |  |  | Unconverted Sequence to analyze | 5’TTCGGGATGGGGACGATGGGGAGGT  1 2  GTCCGAGGTCAAGAGAAAGCGGCACG  3 4 5  AGCAGACCCCTGTGTGCCGT 3’  6 |
|  |  |  | Converted Sequence to analyze (‘-‘ strand) | 5’TTCGGGATGGGGACGATGGGGAGGT  1 2  GTTCGAGGTTAAGAGAAAGCGGTACGA  3 4 5  GTAGATTTTTGTGTGTCG 3’  6 |

Supplemental Table S3.0. Child psychosocial stressors, resilience factors, and methylation correlation table at baseline.

|  | Total Stress | School Stress | IRS | DSS | FPD | SSP | BMI % | WC | *FKBP5* avg | *FKBP5* CpG1 | *FKBP5* CpG2 | *SLC6A4* avg | *SLC6A4* CpG1 | *SLC6A4* CpG2 | *SLC6A4* CpG3 | *SLC6A4* CpG4 | *SLC6A4* CpG5 | *SLC6A4* CpG6 |
| --- | --- | --- | --- | --- | --- | --- | --- | --- | --- | --- | --- | --- | --- | --- | --- | --- | --- | --- |
| Total Stress | 1.00 |  |  |  |  |  |  |  |  |  |  |  |  |  |  |  |  |  |
| School Stress | **0.83** | 1.00 |  |  |  |  |  |  |  |  |  |  |  |  |  |  |  |  |
| IRS | **0.66** | **0.27** | 1.00 |  |  |  |  |  |  |  |  |  |  |  |  |  |  |  |
| DSS | **0.70** | **0.48** | **0.22** | 1.00 |  |  |  |  |  |  |  |  |  |  |  |  |  |  |
| FPD | **0.56** | **0.37** | **0.69** | 0.19 | 1.00 |  |  |  |  |  |  |  |  |  |  |  |  |  |
| SSP | **0.30** | **0.26** | **0.30** | 0.12 | **0.19** | 1.00 |  |  |  |  |  |  |  |  |  |  |  |  |
| BMI % | 0.06 | 0.07 | -0.01 | 0.11 | 0.04 | 0.08 | 1.00 |  |  |  |  |  |  |  |  |  |  |  |
| WC | 0.15 | 0.22 | 0.05 | 0.03 | 0.10 | 0.11 | **0.61** | 1.00 |  |  |  |  |  |  |  |  |  |  |
| *FKBP5* avg | -0.01 | 0.03 | 0.01 | -0.05 | 0.04 | -0.16 | -0.13 | -0.18 | 1.00 |  |  |  |  |  |  |  |  |  |
| *FKBP5* CpG1 | -0.08 | 0.00 | -0.02 | -0.17 | -0.03 | -0.17 | -0.14 | -0.16 | **0.90** | 1.00 |  |  |  |  |  |  |  |  |
| *FKBP5* CpG2 | 0.10 | 0.05 | 0.05 | 0.14 | 0.11 | -0.09 | -0.08 | -0.14 | **0.80** | **0.46** | 1.00 |  |  |  |  |  |  |  |
| *SLC6A4* avg | 0.22 | 0.15 | **0.25** | 0.04 | 0.20 | -0.04 | **-0.39** | **-0.32** | 0.21 | 0.19 | 0.19 | 1.00 |  |  |  |  |  |  |
| *SLC6A4* CpG1 | -0.03 | -0.07 | -0.06 | -0.01 | -0.14 | -0.06 | **-0.28** | **-0.36** | 0.01 | 0.09 | -0.10 | **0.52** | 1.00 |  |  |  |  |  |
| *SLC6A4* CpG2 | 0.16 | 0.09 | 0.19 | 0.04 | 0.12 | -0.04 | -0.21 | -0.18 | 0.10 | 0.12 | 0.04 | **0.78** | **0.45** | 1.00 |  |  |  |  |
| *SLC6A4* CpG3 | 0.22 | 0.17 | **0.29** | 0.03 | 0.22 | 0.05 | **-0.24** | -0.24 | **0.23** | 0.15 | **0.28** | **0.78** | 0.13 | **0.56** | 1.00 |  |  |  |
| *SLC6A4* CpG4 | **0.23** | 0.21 | 0.22 | -0.01 | **0.24** | 0.03 | **-0.25** | -0.11 | 0.17 | 0.12 | 0.19 | **0.73** | 0.10 | **0.49** | **0.54** | 1.00 |  |  |
| *SLC6A4* CpG5 | 0.22 | 0.19 | **0.29** | -0.02 | **0.31** | -0.09 | **-0.29** | -0.20 | **0.25** | 0.20 | **0.25** | **0.82** | 0.08 | **0.58** | **0.77** | **0.64** | 1.00 |  |
| *SLC6A4* CpG6 | **0.26** | 0.15 | **0.28** | 0.15 | **0.25** | -0.03 | **-0.32** | -0.18 | 0.20 | 0.13 | **0.24** | **0.71** | 0.06 | **0.37** | **0.57** | **0.55** | **0.66** | 1.00 |

**Bolded** values represent significant correlations. Key: IRS: immigrant-related stress; DS: discrimination stress; FPD: fear of parent’s deportation; SSP: social support from parents; BMI: body mass index; WC: waist circumference.

Supplemental Table S3.1. Child psychosocial stressors, resilience factors, and methylation correlation table at follow-up.

|  | Total Stress | School Stress | IRS | DSS | FPD | EDS | SSP | YLOT | BMI % | WC | *FKBP5* avg | *FKBP5* CpG1 | *FKBP5* CpG2 | *SLC6A4* avg | *SLC6A4* CpG1 | *SLC6A4* CpG2 | *SLC6A4* CpG3 | *SLC6A4* CpG4 | *SLC6A4* CpG5 | *SLC6A4* CpG6 |
| --- | --- | --- | --- | --- | --- | --- | --- | --- | --- | --- | --- | --- | --- | --- | --- | --- | --- | --- | --- | --- |
| Total Stress | 1.00 |  |  |  |  |  |  |  |  |  |  |  |  |  |  |  |  |  |  |  |
| School Stress | 0.17 | 1.00 |  |  |  |  |  |  |  |  |  |  |  |  |  |  |  |  |  |  |
| IRS | **0.65** | -0.20 | 1.00 |  |  |  |  |  |  |  |  |  |  |  |  |  |  |  |  |  |
| DSS | **0.51** | **0.58** | -0.19 | 1.00 |  |  |  |  |  |  |  |  |  |  |  |  |  |  |  |  |
| FPD | **0.38** | -0.25 | **0.65** | -0.16 | 1.00 |  |  |  |  |  |  |  |  |  |  |  |  |  |  |  |
| EDS | **0.48** | **0.45** | 0.35 | 0.24 | 0.11 | 1.00 |  |  |  |  |  |  |  |  |  |  |  |  |  |  |
| SSP | -0.31 | -0.18 | -0.06 | -0.13 | -0.19 | **-0.46** | 1.00 |  |  |  |  |  |  |  |  |  |  |  |  |  |
| YLOT | -0.12 | -0.20 | -0.11 | 0.10 | -0.13 | **-0.37** | **0.38** | 1.00 |  |  |  |  |  |  |  |  |  |  |  |  |
| BMI % | 0.05 | 0.26 | -0.07 | 0.18 | 0.13 | 0.27 | **-0.36** | -0.18 | 1.00 |  |  |  |  |  |  |  |  |  |  |  |
| WC | 0.25 | 0.13 | 0.17 | 0.00 | 0.11 | **0.40** | -0.09 | -0.26 | 0.32 | 1.00 |  |  |  |  |  |  |  |  |  |  |
| *FKBP5* avg | -0.22 | 0.16 | **-0.43** | 0.29 | -0.29 | **-0.39** | 0.21 | 0.02 | -0.11 | 0.00 | 1.00 |  |  |  |  |  |  |  |  |  |
| *FKBP5* CpG1 | -0.18 | 0.19 | **-0.39** | **0.33** | -0.37 | -0.30 | 0.28 | 0.02 | -0.16 | 0.00 | **0.95** | 1.00 |  |  |  |  |  |  |  |  |
| *FKBP5* CpG2 | -0.24 | 0.10 | **-0.42** | 0.21 | -0.15 | **-0.47** | 0.08 | 0.01 | -0.03 | -0.01 | **0.91** | **0.75** | 1.00 |  |  |  |  |  |  |  |
| *SLC6A4* avg | 0.11 | 0.11 | 0.04 | 0.04 | 0.00 | -0.08 | -0.19 | -0.30 | 0.07 | -0.10 | 0.27 | 0.27 | 0.23 | 1.00 |  |  |  |  |  |  |
| *SLC6A4* CpG1 | 0.17 | -0.01 | 0.01 | 0.12 | -0.05 | -0.07 | -0.14 | **-0.34** | 0.14 | -0.09 | 0.23 | 0.27 | 0.13 | **0.73** | 1.00 |  |  |  |  |  |
| *SLC6A4* CpG2 | 0.02 | 0.02 | -0.01 | -0.01 | 0.03 | -0.14 | -0.32 | -0.27 | -0.09 | -0.15 | 0.31 | 0.32 | 0.26 | **0.83** | **0.53** | 1.00 |  |  |  |  |
| *SLC6A4* CpG3 | 0.14 | 0.00 | 0.12 | 0.04 | 0.13 | -0.18 | -0.20 | 0.00 | 0.08 | -0.16 | 0.11 | 0.06 | 0.17 | **0.78** | **0.37** | **0.59** | 1.00 |  |  |  |
| *SLC6A4* CpG4 | 0.09 | **0.35** | -0.04 | 0.18 | -0.08 | 0.05 | -0.14 | -0.29 | 0.07 | -0.01 | 0.32 | 0.31 | 0.29 | **0.91** | **0.59** | **0.76** | **0.65** | 1.00 |  |  |
| *SLC6A4* CpG5 | 0.01 | 0.22 | -0.02 | -0.04 | -0.01 | -0.03 | -0.01 | **-0.37** | 0.04 | 0.08 | 0.30 | 0.26 | 0.31 | **0.78** | **0.45** | **0.59** | **0.51** | **0.79** | 1.00 |  |
| *SLC6A4* CpG6 | -0.02 | 0.03 | 0.14 | -0.17 | -0.01 | 0.04 | -0.05 | -0.09 | 0.02 | -0.10 | -0.02 | 0.00 | -0.04 | **0.59** | 0.07 | **0.42** | **0.56** | **0.48** | **0.42** | 1.00 |

**Bolded** values represent significant associations. Key: IRS: immigrant-related stress; DS: discrimination stress; FPD: fear of parent’s deportation; EDS: Everyday Discrimination Score; SSP: social support from parents; YLOT: Youth Life Orientation Test; BMI: body mass index; WC: waist circumference.

Supplemental Table S4.0. Maternal psychosocial stressors, resilience factors, and methylation correlation table at baseline.

|  | Total Stress | IRS | DSS | Household Stress | Health Stress | FES | SASH | SSS | SSC | LOT-R | BMI | SBP | DBP | *FKBP5* avg | *FKBP5* CpG1 | *FKBP5* CpG2 | *SLC6A4* avg | *SLC6A4* CpG1 | *SLC6A4* CpG2 | *SLC6A4* CpG3 | *SLC6A4* CpG4 | *SLC6A4* CpG5 | *SLC6A4* CpG6 |
| --- | --- | --- | --- | --- | --- | --- | --- | --- | --- | --- | --- | --- | --- | --- | --- | --- | --- | --- | --- | --- | --- | --- | --- |
| Total Stress | 1.00 |  |  |  |  |  |  |  |  |  |  |  |  |  |  |  |  |  |  |  |  |  |  |
| IRS | **0.74** | 1.00 |  |  |  |  |  |  |  |  |  |  |  |  |  |  |  |  |  |  |  |  |  |
| DSS | **0.63** | **0.45** | 1.00 |  |  |  |  |  |  |  |  |  |  |  |  |  |  |  |  |  |  |  |  |
| Household Stress | **0.72** | **0.35** | **0.31** | 1.00 |  |  |  |  |  |  |  |  |  |  |  |  |  |  |  |  |  |  |  |
| Health Stress | **0.85** | **0.55** | **0.43** | **0.49** | 1.00 |  |  |  |  |  |  |  |  |  |  |  |  |  |  |  |  |  |  |
| FES | **0.87** | **0.50** | **0.50** | **0.69** | **0.59** | 1.00 |  |  |  |  |  |  |  |  |  |  |  |  |  |  |  |  |  |
| SASH | 0.13 | -0.03 | -0.06 | 0.06 | 0.14 | 0.01 | 1.00 |  |  |  |  |  |  |  |  |  |  |  |  |  |  |  |  |
| SSS | **-0.31** | **-0.39** | -0.19 | -0.13 | **-0.30** | -0.15 | -0.06 | 1.00 |  |  |  |  |  |  |  |  |  |  |  |  |  |  |  |
| SSC | -0.13 | -0.11 | 0.03 | -0.10 | -0.18 | -0.02 | -0.13 | **0.29** | 1.00 |  |  |  |  |  |  |  |  |  |  |  |  |  |  |
| LOT-R | -0.13 | -0.21 | 0.16 | **-0.24** | -0.13 | -0.13 | 0.11 | 0.06 | **0.27** | 1.00 |  |  |  |  |  |  |  |  |  |  |  |  |  |
| BMI | 0.01 | 0.00 | -0.02 | -0.06 | 0.09 | -0.08 | 0.01 | -0.09 | **-0.23** | -0.22 | 1.00 |  |  |  |  |  |  |  |  |  |  |  |  |
| SBP | 0.11 | 0.21 | 0.20 | -0.08 | 0.05 | 0.01 | -0.04 | -0.12 | 0.06 | 0.17 | **0.35** | 1.00 |  |  |  |  |  |  |  |  |  |  |  |
| DBP | 0.04 | 0.03 | 0.15 | -0.07 | 0.00 | 0.00 | -0.04 | -0.02 | 0.10 | 0.16 | **0.30** | **0.74** | 1.00 |  |  |  |  |  |  |  |  |  |  |
| *FKBP5* avg | -0.10 | -0.14 | 0.03 | 0.04 | -0.09 | -0.15 | **-0.26** | 0.01 | 0.07 | -0.06 | 0.00 | 0.09 | 0.12 | 1.00 |  |  |  |  |  |  |  |  |  |
| *FKBP5* CpG1 | -0.09 | -0.14 | 0.08 | 0.03 | -0.09 | -0.15 | **-0.29** | 0.07 | 0.07 | -0.12 | 0.04 | 0.08 | 0.11 | **0.96** | 1.00 |  |  |  |  |  |  |  |  |
| *FKBP5* CpG2 | -0.09 | -0.13 | -0.06 | 0.05 | -0.08 | -0.14 | -0.18 | -0.06 | 0.07 | 0.02 | -0.06 | 0.09 | 0.10 | **0.93** | **0.79** | 1.00 |  |  |  |  |  |  |  |
| *SLC6A4* avg | 0.23 | **0.33** | 0.22 | 0.10 | 0.21 | 0.06 | -0.15 | **-0.32** | 0.01 | -0.16 | 0.01 | 0.16 | 0.04 | 0.14 | 0.18 | 0.08 | 1.00 |  |  |  |  |  |  |
| *SLC6A4* CpG1 | **0.24** | **0.32** | **0.23** | 0.12 | 0.15 | 0.09 | -0.22 | **-0.24** | 0.07 | -0.11 | -0.06 | 0.05 | 0.03 | 0.17 | 0.19 | 0.12 | **0.51** | 1.00 |  |  |  |  |  |
| *SLC6A4* CpG2 | 0.15 | **0.23** | 0.17 | 0.12 | 0.11 | 0.06 | -0.17 | -0.17 | 0.09 | -0.09 | -0.03 | 0.17 | 0.12 | 0.13 | 0.14 | 0.10 | **0.86** | **0.28** | 1.00 |  |  |  |  |
| *SLC6A4* CpG3 | 0.18 | **0.26** | 0.16 | -0.01 | **0.25** | 0.03 | -0.03 | **-0.24** | -0.06 | -0.12 | 0.05 | 0.18 | 0.09 | 0.06 | 0.08 | 0.03 | **0.83** | 0.14 | **0.73** | 1.00 |  |  |  |
| *SLC6A4* CpG4 | 0.01 | 0.11 | 0.09 | 0.08 | -0.04 | 0.06 | -0.08 | -0.21 | 0.01 | -0.14 | -0.02 | 0.02 | -0.05 | 0.05 | 0.09 | -0.01 | **0.49** | -0.12 | **0.41** | **0.39** | 1.00 |  |  |
| *SLC6A4* CpG5 | 0.00 | 0.06 | 0.00 | -0.10 | 0.10 | -0.13 | 0.03 | -0.04 | -0.06 | -0.05 | 0.04 | 0.05 | -0.16 | -0.06 | -0.02 | -0.09 | **0.40** | 0.12 | 0.18 | **0.23** | **0.21** | 1.00 |  |
| *SLC6A4* CpG6 | 0.23 | **0.25** | 0.15 | 0.16 | **0.24** | 0.08 | -0.06 | **-0.31** | -0.07 | -0.13 | 0.08 | 0.16 | 0.03 | 0.10 | 0.08 | 0.11 | **0.81** | **0.23** | **0.73** | **0.79** | **0.26** | **0.15** | 1.00 |

**Bolded** values represent significant associations. Key: IRS: immigrant-related stress; DS: discrimination stress; FES: Family Economic Stress; SASH: Short Acculturation Scale for Hispanics; SSS: Subjective Social Status; SSC: social support/social connection; LOT-R: Life Orientation Test – Revised; BMI: body mass index; SBP: systolic blood pressure; DBP: diastolic blood pressure.

Supplemental Table S4.1. Maternal psychosocial stressors, resilience factors, and methylation correlation table at follow-up.

|  | Total Stress | IRS | DSS | Household Stress | Health Stress | FES | EDS | SASH | SSS | SSC | LOT-R | BMI | SBP | DBP | *FKBP5* avg | *FKBP5* CpG1 | *FKBP5* CpG2 | *SLC6A4* avg | *SLC6A4* CpG1 | *SLC6A4* CpG2 | *SLC6A4* CpG3 | *SLC6A4* CpG4 | *SLC6A4* CpG5 | *SLC6A4* CpG6 |
| --- | --- | --- | --- | --- | --- | --- | --- | --- | --- | --- | --- | --- | --- | --- | --- | --- | --- | --- | --- | --- | --- | --- | --- | --- |
| Total Stress | 1.00 |  |  |  |  |  |  |  |  |  |  |  |  |  |  |  |  |  |  |  |  |  |  |  |
| IRS | **0.54** | 1.00 |  |  |  |  |  |  |  |  |  |  |  |  |  |  |  |  |  |  |  |  |  |  |
| DSS | **0.61** | 0.17 | 1.00 |  |  |  |  |  |  |  |  |  |  |  |  |  |  |  |  |  |  |  |  |  |
| Household Stress | **0.39** | 0.14 | 0.13 | 1.00 |  |  |  |  |  |  |  |  |  |  |  |  |  |  |  |  |  |  |  |  |
| Health Stress | **0.65** | **0.38** | 0.19 | **0.43** | 1.00 |  |  |  |  |  |  |  |  |  |  |  |  |  |  |  |  |  |  |  |
| FES | **0.79** | 0.16 | **0.48** | 0.21 | 0.00 | 1.00 |  |  |  |  |  |  |  |  |  |  |  |  |  |  |  |  |  |  |
| EDS | **0.40** | 0.16 | **0.67** | 0.12 | -0.01 | **0.59** | 1.00 |  |  |  |  |  |  |  |  |  |  |  |  |  |  |  |  |  |
| SASH | -0.08 | 0.06 | 0.06 | -0.10 | -0.12 | -0.08 | 0.19 | 1.00 |  |  |  |  |  |  |  |  |  |  |  |  |  |  |  |  |
| SSS | -0.11 | -0.10 | -0.19 | 0.11 | 0.09 | -0.10 | -0.02 | -0.11 | 1.00 |  |  |  |  |  |  |  |  |  |  |  |  |  |  |  |
| SSC | -0.06 | 0.14 | -0.17 | -0.13 | 0.05 | -0.17 | -0.19 | -0.02 | 0.18 | 1.00 |  |  |  |  |  |  |  |  |  |  |  |  |  |  |
| LOT-R | 0.06 | -0.06 | 0.25 | -0.11 | -0.15 | 0.16 | **0.34** | 0.16 | -0.10 | -0.05 | 1.00 |  |  |  |  |  |  |  |  |  |  |  |  |  |
| BMI | 0.02 | -0.04 | 0.06 | 0.12 | 0.16 | -0.15 | -0.19 | **-0.30** | -0.12 | -0.03 | -0.16 | 1.00 |  |  |  |  |  |  |  |  |  |  |  |  |
| SBP | 0.15 | -0.06 | 0.27 | -0.25 | 0.14 | -0.15 | -0.13 | 0.07 | **-0.32** | 0.07 | 0.07 | 0.37 | 1.00 |  |  |  |  |  |  |  |  |  |  |  |
| DBP | 0.02 | -0.03 | 0.18 | -0.18 | -0.02 | -0.20 | -0.09 | 0.25 | -0.27 | 0.25 | -0.05 | 0.23 | **0.64** | 1.00 |  |  |  |  |  |  |  |  |  |  |
| *FKBP5* avg | -0.09 | -0.15 | 0.02 | -0.03 | -0.13 | 0.12 | 0.07 | 0.03 | -0.05 | -0.05 | -0.05 | 0.07 | -0.01 | -0.05 | 1.00 |  |  |  |  |  |  |  |  |  |
| *FKBP5* CpG1 | -0.16 | -0.21 | 0.06 | -0.05 | -0.20 | 0.11 | 0.17 | 0.07 | -0.07 | -0.09 | -0.01 | -0.03 | 0.00 | -0.01 | **0.94** | 1.00 |  |  |  |  |  |  |  |  |
| *FKBP5* CpG2 | 0.00 | -0.05 | -0.03 | -0.01 | -0.04 | 0.11 | -0.07 | -0.02 | -0.01 | -0.01 | -0.09 | 0.17 | -0.01 | -0.08 | **0.92** | **0.74** | 1.00 |  |  |  |  |  |  |  |
| *SLC6A4* avg | -0.16 | -0.05 | 0.04 | -0.13 | -0.13 | -0.10 | 0.11 | **0.37** | 0.04 | 0.06 | 0.04 | -0.06 | -0.04 | **0.45** | -0.05 | 0.04 | -0.14 | 1.00 |  |  |  |  |  |  |
| *SLC6A4* CpG1 | -0.10 | 0.12 | -0.08 | -0.05 | -0.25 | 0.07 | 0.21 | 0.21 | 0.16 | 0.19 | 0.04 | -0.12 | -0.24 | 0.01 | **0.35** | **0.36** | 0.29 | **0.57** | 1.00 |  |  |  |  |  |
| *SLC6A4* CpG2 | -0.17 | 0.06 | -0.03 | 0.00 | -0.07 | -0.09 | 0.06 | 0.28 | 0.08 | 0.19 | -0.12 | -0.14 | -0.14 | **0.43** | -0.08 | 0.01 | -0.16 | **0.90** | **0.47** | 1.00 |  |  |  |  |
| *SLC6A4* CpG3 | -0.13 | -0.10 | 0.12 | -0.13 | -0.11 | -0.10 | 0.09 | 0.31 | 0.05 | 0.03 | 0.03 | 0.02 | 0.00 | **0.43** | -0.15 | -0.04 | -0.25 | **0.92** | **0.40** | **0.80** | 1.00 |  |  |  |
| *SLC6A4* CpG4 | -0.37 | -0.25 | -0.03 | **-0.34** | -0.25 | -0.24 | -0.01 | **0.38** | -0.12 | 0.01 | -0.03 | -0.04 | 0.06 | **0.51** | -0.01 | 0.10 | -0.14 | **0.86** | **0.33** | **0.72** | **0.86** | 1.00 |  |  |
| *SLC6A4* CpG5 | 0.12 | 0.03 | 0.26 | -0.08 | 0.08 | -0.05 | 0.15 | **0.35** | -0.04 | -0.08 | 0.12 | 0.14 | 0.18 | **0.48** | -0.22 | -0.18 | -0.23 | **0.78** | 0.16 | **0.61** | **0.70** | **0.66** | 1.00 |  |
| *SLC6A4* CpG6 | -0.11 | -0.11 | 0.02 | -0.03 | 0.03 | -0.10 | 0.00 | 0.26 | 0.05 | -0.06 | 0.13 | -0.12 | 0.01 | **0.40** | -0.29 | -0.21 | **-0.34** | **0.83** | 0.17 | **0.78** | **0.77** | **0.66** | **0.76** | 1.00 |

**Bolded** values represent significant associations. Key: IRS: immigrant-related stress; DS: discrimination stress; FES: Family Economic Stress; EDS: Everyday Discrimination Score; SASH: Short Acculturation Scale for Hispanics; SSS: Subjective Social Status; SSC: social support/social connection; LOT-R: Life Orientation Test – Revised; BMI: body mass index; SBP: systolic blood pressure; DBP: diastolic blood pressure.
